# Effects of Wall Temperature on Scalar and Turbulence Statistics During Premixed Flame–Wall Interaction Within Turbulent Boundary Layers

**DOI:** 10.1007/s10494-024-00603-w

**Published:** 2025-01-16

**Authors:** Sanjeev Kr. Ghai, Umair Ahmed, Nilanjan Chakraborty

**Affiliations:** 1 School of Engineering, Newcastle University, Newcastle-Upon-Tyne, NE1 7RU UK; 2 Department of Engineering, University of Cambridge, Cambridge, CB21PZ UK

**Keywords:** Premixed flame–wall interaction, Thermal boundary condition, Scalar variance, Scalar dissipation rate, Reynolds stress, Direct numerical simulations

## Abstract

Direct numerical simulations (DNS) have been utilised to investigate the impact of different thermal wall boundary conditions on premixed V-flames interacting with walls in a turbulent channel flow configuration. Two boundary conditions are considered: isothermal walls, where the wall temperature is set either equal to the unburned mixture temperature or an elevated temperature, and adiabatic walls. An increase in wall temperature has been found to decrease the minimum flame quenching distance and increase the maximum wall heat flux magnitude. The analysis reveals notable differences in mean behaviours of the progress variable and non-dimensional temperature in response to thermal boundary conditions. At the upstream of the flame–wall interaction location, higher mean friction velocity values are observed for the case with elevated wall temperature compared to the other cases. However, during flame–wall interaction, friction velocity values decrease for isothermal walls but initially rise before decreasing for adiabatic walls, persisting at levels surpassing isothermal conditions. For all thermal wall boundary conditions, the mean scalar dissipation rates of the progress variable and non-dimensional temperature exhibit a decreasing trend towards the wall. Notably, in the case of isothermal wall boundary condition, a higher scalar dissipation rate for the non-dimensional temperature is observed in comparison to the scalar dissipation rate for the progress variable. Thermal boundary condition also has a significant impact on Reynolds stress components, turbulent kinetic energy, and dissipation rates, showing the highest magnitudes with isothermal case with elevated wall temperature and the lowest magnitude for the isothermal wall with unburned gas temperature. The findings of the current analysis suggest that thermal boundary conditions can potentially significantly affect trubulence closures in the context of Reynolds averaged Navier–Stokes simulations of premixed flame–wall interaction.

## Introduction

The premixed flame–wall interaction (FWI) in turbulent boundary layers has been the focus of several experimental (e.g., Dreizler and Böhm 2015; Jainski et al. 2017, 2018; Johe et al. 2022; Kosaka et al. 2018, 2020; Mann et al. 2014; Ojo et al. 2021, 2022; Renaud et al. 2018; Zentgraf et al. 2021, 2022, 2024) and numerical (Ahmed et al. 2021a, b, c, 2023; Alshalaan and Rutland 1998, 2002; Bruneaux et al. 1996, 1997; Ghai et al. 2022a, b; 2023a, b, 2023c; Gruber et al. 2010; Jiang et al. 2019, 2021; Kaddar et al. 2023; Kai et al. 2023; Paluli et al. 2019; Steinhausen et al. 2023) investigations. This is motivated by the miniaturisation of combustors to increase the power-density and make them compatible with electrical drives. However, a decrease in combustor size increases the likelihood of FWI. The heat loss through the combustor walls leads to flame quenching (Poinsot et al. 1993; Alshalaan and Rutland 2002; Gruber et al. 2010; Lai and Chakraborty 2016a; Ghai et al. 2022b, 2023b) and determines the cooling requirements in order to maintain the structural integrity of combustor walls. The flame is more likely to quench for a higher surface area to volume ratio for miniaturised combustors, which often limits the usage of micro-combustors. Thus, it is important to analyse the impact of the conditions prevailing at the wall on the flame–wall interaction, quenching distance and the underlying turbulent flow statistics within the boundary layer during premixed FWI. Although previous studies on premixed FWI within turbulent boundary layers focussed on various aspects of flame structure (Ahmed et al. 2021a; Gruber et al. 2010; Kosaka et al. 2018; Johe et al. 2022; Kai et al. 2023; Kosaka et al. 2020; Kaddar et al. 2023); the role of flame-vortex interaction (Ghai et al. 2023b; Steinhausen et al. 2023); turbulent flow statistics (Ahmed et al. 2021b, 2023; Ghai et al. 2022a); and wall heat flux, flame quenching and heat transfer characteristics (Alshaalan and Rutland 2002; Ghai et al. 2022b, 2023b; Gruber et al. 2010; Lai and Chakraborty, 2016a; Ojo et al. 2021, 2022; Poinsot et al. 1993), limited attention was given to the effects of thermal wall boundary condition on premixed FWI within turbulent boundary layers (Ahmed et al. 2021a,b, 2023; Ghai et al. 2022a, 2023a).

Ahmed et al. (2023) analysed the turbulent kinetic energy, Reynolds stress and dissipation rate of turbulent kinetic energy statistics for head-on and oblique wall interactions of premixed flames within turbulent boundary layers for both isothermal and adiabatic wall boundary conditions using DNS data. The same DNS database was utilised to analyse the statistics of scalar variances, turbulent scalar fluxes and scalar dissipation rates of reaction progress variable and non-dimensional temperature for both isothermal and adiabatic wall boundary conditions by Ahmed et al. (2021b). The statistics of the reactive scalar gradient for oblique wall interaction of V-shaped premixed turbulent flames were analysed by Ahmed et al. (2021a) in a channel flow configuration for both isothermal and adiabatic wall boundary conditions. The effects of wall boundary conditions on turbulent kinetic energy and enstrophy transport for head-on interaction of statistically planar premixed turbulent flame propagating across the turbulent boundary layer have been analysed by Ghai et al. (2022a, 2023a). The aforementioned analyses revealed that the heat loss through the wall for isothermal boundary conditions leads to flame quenching, whereas the flame does not quench for adiabatic boundary conditions and chemical reaction rate occurs at the wall. This affects the scalar variance, turbulent scalar flux and scalar dissipation rate (i.e. scalar gradient) evolutions close to the wall (Ahmed et al. 2021a, b). However, the effects of elevated temperature on FWI have not been analysed in detail. Zhao et al. (2018) considered the effects of elevated wall temperature on the flame quenching and wall heat flux statistics for statistically planar flames impinging on inert isothermal walls, but that analysis did not include any conventional boundary layer effects. This gap in the existing literature is addressed in this analysis by considering a direct numerical simulations (DNS) database of oblique flame–wall interaction of V-shaped turbulent premixed flames in a turbulent channel flow configuration for different thermal wall boundary conditions. In this respect, the main objectives of this paper are as follows:

- To demonstrate the influence of wall temperature on wall heat stress, wall heat flux and quenching distance during premixed FWI in turbulent boundary layers.
- To indicate the effects of wall temperature on the distributions of turbulence (e.g., Reynolds stresses, turbulent kinetic energy and its dissipation rate) and reactive scalar (Reynolds scalar flux, variance and dissipation rates) statistics during oblique wall interaction of V-shaped premixed flames in turbulent boundary layers.
- To provide physical explanations for (a) and (b).

The remainder of the paper is organised as follows. The necessary mathematical background and the information related to the DNS database considered in this analysis are provided in the next section. This is followed by the presentation of the results and their discussion. The main findings are summarised and conclusions are drawn in the final section of this paper.

## Mathematical background and numerical implementation

The reacting scalar field in premixed turbulent premixed flames is characterised in terms of reaction progress variable $c$ and non-dimensional temperature $\theta$ in the following manner:

1 $$ c = \left( {Y - Y_{u} } \right)/\left( {Y_{b} - Y_{u} } \right)\;{\text{and}}\;\theta = \left( {T - T_{u} } \right)/\left( {T_{ad} - T_{u} } \right) $$

where $Y$ is the mass fraction of appropriate species (which is taken to be the fuel mass fraction for the current analysis) and the subscripts $u$ and $b$ refer to values in the unburned gas and fully burned products, respectively and ${T}_{ad}$ is the adiabatic flame temperature. The modelling of non-adiabatic flows involves the knowledge of $\widetilde{c}$ and $\widetilde{\theta }$ (where $\widetilde{q}=\overline{\rho q }/\overline{\rho }$ is the Favre-averaged value of a general variable $q$ with $\rho$ and $\overline{q }$ being the density and the Reynolds averaged value of $q$, respectively) along with the corresponding variances (i.e., $\widetilde{{c}^{{\prime}{\prime}2}}$ and $\widetilde{{\theta }^{{\prime}{\prime}2}}$ where ${q}^{{\prime}{\prime}}=q-\widetilde{q}$ is the Favre fluctuation of $q$) (Libby and Williams 1983).

The transport equations of $\widetilde{c}$ and $\widetilde{\theta }$ take the following form (Poinsot and Veynante 2001):

2i $$\frac{\partial (\overline{\rho }\widetilde{c})}{\partial t}+\frac{\partial (\overline{\rho }{\widetilde{u}}_{j}\widetilde{c})}{\partial {x}_{j}}=\frac{\partial }{\partial {x}_{j}}\left(\overline{\rho }\widetilde{D}\frac{\partial \widetilde{c}}{\partial {x}_{j}}\right)-\frac{\partial \left(\overline{\rho {u }_{j}^{{\prime}{\prime}}{c}^{{\prime}{\prime}}}\right)}{\partial {x}_{j}}+{\dot{\omega }}_{c}$$

2ii $$ \frac{{\partial \left( {\overline{\rho }\tilde{\theta }} \right)}}{\partial t} + \frac{{\partial \left( {\overline{\rho }\tilde{u}_{j}^{{\tilde{\theta }}} } \right)}}{{\partial x_{j} }} = \frac{\partial }{{\partial x_{j} }}\left( {\overline{\rho }\tilde{\alpha }_{T} \frac{{\partial \tilde{\theta }}}{{\partial x_{j} }}} \right) - \frac{{\partial \left( {\overline{{\rho u_{j}^{{^{\prime\prime}}} \theta^{\prime\prime}}} } \right)}}{{\partial x_{j} }} + \dot{\omega }_{\theta } $$

where $\rho$ is the gas density, ${u}_{j}$ is the j^th^ component of fluid velocity, ${\alpha }_{T}$ is the thermal diffusivity, $D$ is the reaction progress variable diffusivity, ${\dot{\omega }}_{c}$ is the reaction progress variable reaction rate and ${\dot{\omega }}_{\theta }$ is the heat release rate contribution in the non-dimensional temperature equation. In Eqs. 2i and 2ii, $\left(\overline{\rho {u }_{j}^{{\prime}{\prime}}{c}^{{\prime}{\prime}}}\right)$ and $\left(\overline{\rho {u }_{j}^{{\prime}{\prime}}{\theta }^{{\prime}{\prime}}}\right)$ are Reynolds fluxes of reaction progress variable and non-dimensional temperature, respectively. In these equations, both mean reaction/heat release rate and turbulent scalar flux terms are unclosed and their behaviours in response to wall temperature variations are yet to be analysed, which will be addressed in the next section.

Moreover, both $\widetilde{{c}^{{\prime}{\prime}2}}$ and $\widetilde{{\theta }^{{\prime}{\prime}2}}$ are also unclosed terms and their transport equations take the following form (Chakraborty and Swaminathan 2010; Ghai et al. 2024):

3i $$\frac{\partial (\overline{\rho }\widetilde{{c}^{{\prime}{\prime}2}})}{\partial t}+\frac{\partial (\overline{\rho }{\widetilde{u}}_{j}\widetilde{{c}^{{\prime}{\prime}2}})}{\partial {x}_{j}}=\frac{\partial }{\partial {x}_{j}}\left[\overline{\rho }\widetilde{D}\frac{\partial \widetilde{{c}^{{\prime}{\prime}2}}}{\partial {x}_{j}}\right]-\frac{\partial \left({\overline{\rho {u}_{j}^{{\prime}{\prime}}{{{c}^{{\prime}{\prime}}}^{2}}}}\right)}{\partial {x}_{j}}-2\overline{\rho {u}_{j}^{{\prime}{\prime}}{{{c}^{{\prime}{\prime}}}}}\frac{\partial \widetilde{c}}{\partial {x}_{j}}+2\left(\overline{\dot{{\omega  }_{c}}c}-\overline{\dot{{\omega  }_{c}}}\widetilde{c}\right)-2\overline{\rho }{\widetilde{\varepsilon }}_{c}$$

3ii $$ \begin{aligned}    & \frac{{\partial \left( {\bar{\rho }\widetilde{{\theta ^{{\prime \prime 2}} }}} \right)}}{{\partial t}} + \frac{{\partial \left( {\bar{\rho }\tilde{u}_{j} \theta \widetilde{{^{{\prime \prime 2}} }}} \right)}}{{\partial x_{j} }} \\     &  = \frac{\partial }{{\partial x_{j} }}\left[ {\bar{\rho }\tilde{\alpha }_{T} \frac{{\partial \widetilde{{\theta ^{{\prime \prime 2}} }}}}{{\partial x_{j} }}} \right] -  \frac{{\partial \left( {\overline{{\rho u_{j}^{{\prime \prime }} \theta ^{{\prime \prime 2}} }} } \right)}}{{\partial x_{j} }}  - 2\overline{{\rho u_{j}^{{\prime \prime }} \theta ^{{\prime \prime }} }} \frac{{\partial \tilde{\theta }}}{{\partial x_{j} }} + 2\left( {\overline{{\dot{\omega }_{\theta } \theta }}  - \overline{{\dot{\omega }_{\theta } }} \tilde{\theta }} \right) - 2\bar{\rho }\tilde{\varepsilon }_{\theta }  \\  \end{aligned}  $$

The closures of unclosed terms of Eqs. 3i and 3ii with and without FWI have been discussed elsewhere (Lai and Chakraborty 2016c; Ghai et al. 2024) and these analyses revealed that the statistical behaviours and closures of $\overline{\rho {u}_{j}^{{\prime}{\prime}}{{{c}^{{\prime}{\prime}}}^{2}}}$ and ${\overline{\rho {u}_{j}^{{\prime}{\prime}}{{{\theta }^{{\prime}{\prime}}}^{2}}}}$ depend on $\overline{\rho {u}_{j}^{{\prime}{\prime}}{{c}^{{\prime}{\prime}}}}$ and ${\overline{\rho {u}_{j}^{{\prime}{\prime}}{{\theta }^{{\prime}{\prime}}}}}$, respectively. Moreover, ${\widetilde{\varepsilon }}_{c}=\overline{\rho D\nabla {c }^{{\prime}{\prime}}\bullet \nabla {c}^{{\prime}{\prime}}}/\overline{\rho }$ and ${\widetilde{\varepsilon }}_{\theta }=\overline{\rho D\nabla {\theta  }^{{\prime}{\prime}}\bullet \nabla {\theta }^{{\prime}{\prime}}}/\overline{\rho }$, which are scalar dissipation rates of reaction progress variable and non-dimensional temperature, respectively, are unclosed terms and they are also important for the closures of $2\left(\overline{{\dot{\omega } }_{c}c}-\overline{{\dot{\omega } }_{c}}\widetilde{c}\right)$ and$2\left(\overline{{\dot{\omega } }_{\theta }\theta }-\overline{{\dot{\omega } }_{\theta }}\widetilde{\theta }\right)$, respectively (Chakraborty and Swaminathan 2010; Ghai et al. 2024). Once again, the implications of wall boundary conditions on the scalar dissipation rates of reaction progress variable and non-dimensional temperature are addressed in Sect. 3 of this paper.

Finally, flame quenching due to wall heat loss through the cold wall in FWI is characterised in terms of normalised wall heat flux ${\Phi }_{w}$ and Pectlet number $Pe$ (Huang et al. 1988; Jarosinski 1986; Lai & Chakraborty 2016a,b; Poinsot et al. 1993; Vosen et al. 1985):

4 $$ {\Phi }_{w} = |q_{w} |/\left[ {\rho_{0} c_{p0} S_{L} \left( {T_{ad} - T_{u} } \right)} \right]\;{\text{and}}\;Pe = y_{{\theta^{*} }} /\delta_{z} $$

Here. ${{q}_{w},\rho }_{0}$, ${c}_{p0}$ and ${S}_{L}$ are the wall heat flux, unburned gas density, unburned gas specific heat at constant pressure, unstretched laminar burning velocity, unburned gas temperature, and adiabatic flame temperature, respectively. In Eq. 4, ${y}_{\theta }^{*}$ is the wall-normal distance of a given non-dimensional temperature, ${\theta }^{*}$, isosurface such that the maximum heat release rate occurs in the unstretched laminar premixed flame at $\theta ={\theta }^{*}$ and ${\delta }_{z}={\alpha }_{T0}/{S}_{L}$ signifies the Zel’dovich flame thickness where ${\alpha }_{T0}=\lambda /{\rho }_{0}{c}_{p0}$ is the thermal diffusivity of the unburned gas. The wall heat flux ${q}_{w}$ is defined as: ${q}_{w}=-\lambda {\left(\partial T/\partial y\right)}_{y=0}$ with $\lambda$ being the thermal conductivity. Equation 4 suggests that the minimum value of the Peclet number yields the measure of the minimum flame quenching distance ${\delta }_{Q}$ in the following manner:

5 $$P{e}_{min}={\delta }_{Q}/{\delta }_{z}$$

The effects of wall temperature on ${\Phi }_{w}$ and $P{e}_{min}$ are also analysed in the next section of this paper.

In order to conduct a detailed parametric analysis in terms of wall boundary condition, the chemical reaction is simplified by a single-step irreversible reaction given by: $1.0 \;\text{unit\; mass of Fuel}+s \;\text{unit\; mass of Oxidiser}\to \left(1+s\right) \text\;{unit\; mass of Products}$ (where $s$ is the stoichiometric ratio of oxidiser to fuel by mass) for the purpose of computational economy. For all the cases, stoichiometric methane-air mixture (i.e., $s=4.0$) preheated to 730 K (i.e., the unburned gas temperature) has been considered under atmospheric conditions, which yields a heat release parameter $\tau =({T}_{ad}-{T}_{u})/{T}_{u}$ of 2.3 (i.e., $\tau =2.3$). The Prandtl number $Pr$ and the ratio of specific heats $\gamma$ are taken to be 0.7 and 1.4, respectively (i.e., $Pr=0.7$ and $\gamma =1.4$) and Lewis number is taken to be unity for all the simulations. Several previous analyses (Lai et al. 2018; Zhao et al. 2023) demonstrated that the statistical behaviours of wall heat flux and flame quenching distance obtained from single-step chemistry DNS (Lai and Chakraborty 2016a,b; Poinsot et al. 1993) are found to be consistent with the results from detailed chemistry DNS data (Lai et al. 2018; Zhao et al. 2023) and experimental findings (Huang et al. 1988; Jarosinski 1986; Vosen et al. 1985). Moreover, the mean reaction rate closures based on Flame Surface Density (FSD) and scalar dissipation rate (SDR) methodologies developed using single-step chemistry DNS (Lai and Chakraborty 2016a,b; Sellmann et al. 2017) have been found to be valid in the presence of detailed chemistry (Lai et al. 2018, 2022). This suggests that the findings of the current analysis will at least be valid in a qualitative sense in comparison to results obtained from DNS with complex chemistry and transport.

The simulations for the current analysis have been conducted using a three-dimensional compressible DNS code called SENGA + (Jenkins & Cant 1999). In SENGA + , the conservation equations of mass, momentum, energy and species are solved in non-dimensional form, and all the spatial derivatives for the internal grid points have been approximated by a 10th order central difference scheme but a gradual drop of the order of accuracy occurs towards the non-periodic boundaries where a one-sided 2nd order scheme is used. An explicit low-storage 3rd order Runge–Kutta scheme is used for time-advancement. An incompressible non-reacting turbulent channel flow with flow properties corresponding to the unburned gas temperature ${T}_{u}$ subjected to a constant streamwise pressure gradient (i.e. $-\partial p/\partial x=\rho {\overline{u} }_{\tau ,NR}^{2}/h$ where $p$ is the pressure, ${\overline{u} }_{\tau ,NR}=\sqrt{\left|{\overline{\tau }}_{w.NR}\right|/\rho }$ is the friction velocity for the non-reacting incompressible channel flow, ${\overline{\tau }}_{w,NR}$ is the mean wall shear stress for the non-reacting channel flow and $h$ is the channel half height) is considered for the initialisation of the velocity field and also for the purpose of inflow boundary condition specification. The friction Reynolds number $R{e}_{\tau }={\rho }_{0}{u}_{\tau ,NR}h/{\mu }_{0}$ is taken to be 110 for this non-reacting flow simulation, which is comparable to the value used in several recent experimental investigations (Zentgraf et al. 2021, 2022, 2024). This non-reacting channel flow has a bulk Reynolds number of $R{e}_{b}={2\rho }_{NR}{u}_{b}h/{\mu }_{NR}=3285$ where ${u}_{b}=1/2h{\int }_{0}^{2h}udy$ is the bulk mean velocity, ${\rho }_{NR}$ is the unburned gas density and ${\mu }_{NR}$ is the unburned gas viscosity. The ratio of unstretched laminar burning velocity to the non-reacting flow friction velocity (i.e. ${S}_{L}/{\overline{u} }_{\tau ,NR}$) is taken to be 0.7 for all cases considered here. It was demonstrated elsewhere (Ahmed et al. 2021a,b) that both mean and fluctuating velocity statistics for the non-reacting flow simulation remain in excellent agreement with previous DNS results (Tsukahara et al. 2005). For $R{e}_{\tau }=110$ channel flow, the longitudinal integral length scale ${L}_{11}$ scales with $h$, and the root-mean-square velocity fluctuation scales with ${\overline{u} }_{\tau ,NR}$ (Ahmed et al. 2021d), which yields a Damköhler number $Da={L}_{11}{S}_{L}/{u}^{\prime}{\delta }_{th}$ of 15.80 and a Karlovitz number $Ka={\left({u}^{\prime}/{S}_{L}\right)}^{3/2}{\left({L}_{11}/{\delta }_{th}\right)}^{-1/2}$) of 0.36. These values are representative of the corrugated flamelets regime combustion when the flame is away from the wall (Peters 2000).

The simulation domain is taken to be ${L}_{x}\times {L}_{y}\times {L}_{z}=22.22h\times 2h\times 4h$ for all the simulations, and it is discretised using an equidistant Cartesian grid of $4000\times 360\times 720$. This grid ensures 8 points within the thermal flame thickness ${\delta }_{th}=({T}_{ad}-{T}_{0})/{\text{max}\left|\nabla \widehat{T}\right|}_{L}$ and the maximum value of ${y}^{+}={\rho }_{NR}{u}_{\tau NR}y/{\mu }_{NR}$ for the grid points adjacent to the wall remains smaller than 0.6. As Karlovitz number scales as $Ka\sim {\delta }_{th}^{2}/{\eta }^{2}$ (where $\eta$ is the Kolmogorov length scale), the grid spacing is much smaller than the Kolmogorov length scale. According to Tennekes and Lumley (1972), one gets ${y}^{+}<{\eta }^{+}={\rho }_{NR}{u}_{\tau NR}\eta /{\mu }_{NR}$ for ${y}^{+}<1.0$, whereas ${y}^{+}>{\eta }^{+}$ is obtained for ${y}^{+}>1.0$. Thus, a grid spacing which yields ${y}^{+}=0.6$ also ensures that the Kolmogorov length scale is resolved in the vicinity of the wall.

For these V-flame simulations, the flame holder is placed within the fully developed channel flow at a location such that the centre of the flame holder resides at a streamwise distance of ${x}_{h}=0.83h$ from the inlet and a wall normal distance of $h$ (i.e., corresponds to ${y}^{+}=110$ for the non-reacting flow) from the bottom wall. The radius of the flame holder is taken to be $0.5{\delta }_{th}$ and have been implemented following the practice of Dunstan et al. (2010). A time dependent fully developed turbulent boundary layer inflow with specified density and velocity components from non-reacting flow simulations is specified on the left-hand side boundary in the streamwise direction (i.e., $x-$direction). The right-hand side boundary in $x-$ direction is taken to be a partially non-reflecting outflow and is specified according to the Navier–Stokes Characteristic Boundary Conditions (NSCBC) by Yoo and Im (2007). For all the simulations, the walls are taken to be chemically inert, no-slip and impenetrable. Three different thermal boundary conditions have been considered for the walls. In case A, the walls are considered to be isothermal and wall temperatures are taken to be the same as that of the unburned gas temperature (i.e., ${\theta }_{y=0}={\theta }_{y=2h}=0)$. For the purpose of comparison, an additional case (i.e., case B) is considered where the walls are considered to be adiabatic (i.e., ${(\partial \theta /\partial y)}_{y=0}={(\partial \theta /\partial y)}_{y=2h}=0$). Isothermal walls are also considered in case C, but the walls are kept at an elevated temperature (i.e., ${\theta }_{y=0}={\theta }_{y=2h}=0.3)$. The spanwise direction is considered to be periodic for all cases considered here. The simulation has been conducted for one flow through time to allow for the decay of initial transience and followed subsequently for $2.0$ flow through times based on bulk velocity (i.e., $2.0{L}_{x}/{u}_{b}$). The time-averaging over the final $2.0$ flow through times and subsequently by spatial averaging in the statistically homogeneous $z-$ direction have been employed to obtain the Reynolds/Favre averaged quantities.

## Results and discussion

### Wall Boundary Condition Effects on the Evolution of Flame–Wall Interaction

**Fig. 1:**
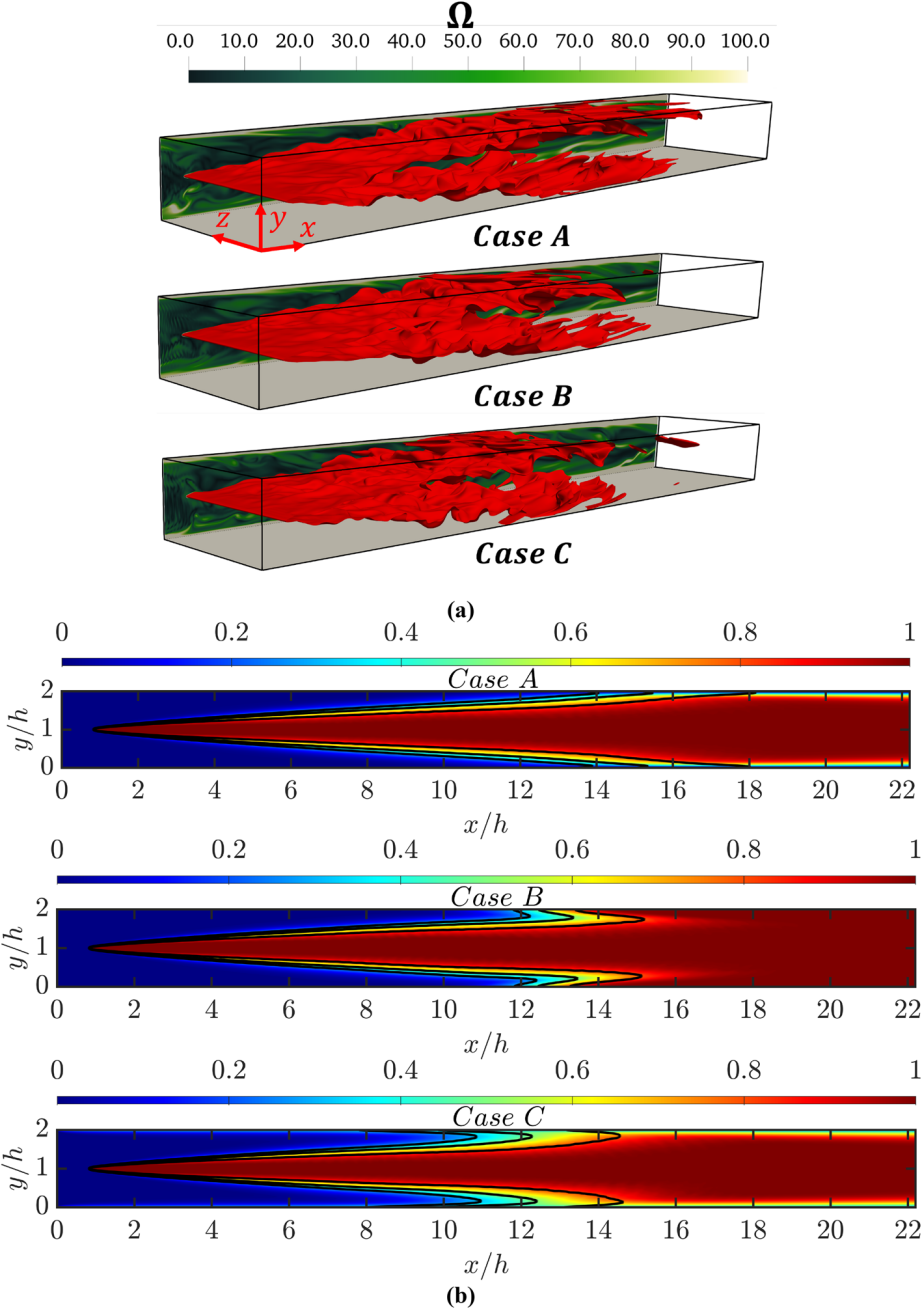
**a** Isosurfaces of $c=0.5$ (shown in red) after reaching a statistically steady state for oblique wall-interaction of turbulent V-flames for cases A (i.e. isothermal wall ${\theta }_{w}=0$), B (i.e., adiabatic wall) and C (i.e. isothermal wall ${\theta }_{w}=0.3$). The distributions of normalised vorticity magnitude $\Omega =\sqrt{{w}_{i}{w}_{i}}\times h/{\overline{u} }_{\tau ,NR}$ at $z/h=4.0$ are also shown. **b** Fields of Favre-averaged temperature $\widetilde{\theta }$ with $\widetilde{c}=\text{0.3,0.5},$ and 0.8 (shown in black) superimposed for oblique wall-interaction of turbulent V-flames for cases A-C.

**Fig. 2:**
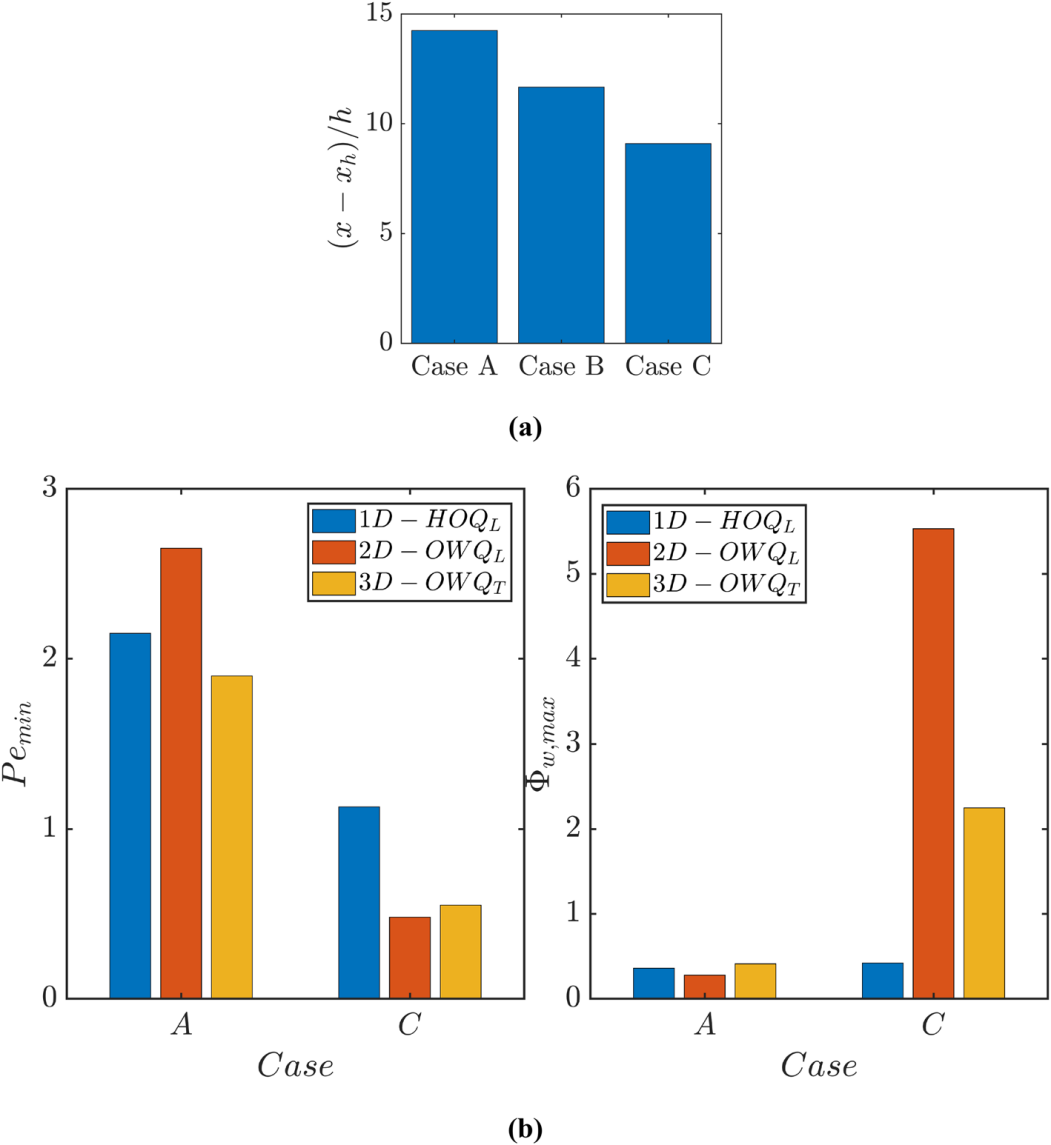
**a** Streamwise distance from the flame holder $(x-{x}_{h})/h$ of the intersection point of $\widetilde{c}=0.5$ contour with the wall and **b** Values of the minimum Peclet number $P{e}_{min}$ and maximum values of normalised wall heat flux magnitude ${\Phi }_{w,max}$

The instantaneous views of the reaction progress variable $c=0.5$ isosurfaces for cases A-C are shown in Fig. 1a where the distributions of normalised vorticity magnitude $\Omega =\sqrt{{w}_{i}{w}_{i}}\times h/{u}_{\tau ,NR}$ (where ${w}_{i}$ is the *i*^*th*^ component of vorticity) at $z/h=4.0$ are shown. It can be appreciated from Fig. 1a that the near wall vortical structures influence the flame wrinkling, which affects the nature of FWI in all cases considered here. The distributions of Favre-averaged non-dimensional temperature $\widetilde{\theta }$ are shown in Fig. 1b where $\widetilde{c}=\text{0.3,0.5}$ and 0.8 contours are also superimposed for cases A-C. It can be seen from Fig. 1 that the flame starts to interact with the wall at $x/h>12$ for case A, but it can be seen clearly from Fig. 1b that the flame tends to move upstream close to the wall in the region where the upcoming fluid moves relatively slowly in cases B and C. Therefore, the flame in cases C and B start to interact with the wall closer to the flame holder than in case A. This is illustrated quantitatively in Fig. 2a where the variation of normalised streamwise distance from the flame holder $(x-{x}_{h})/h$ of the intersection point of the $\widetilde{c}=0.5$ contours with the wall is presented. Figure 2a shows that the intersection of the flame with the wall occurs at the highest value of $(x-{x}_{h})/h$ for case A where the wall temperature is kept at the same temperature as that of the unburned gas mixture. The intersection of the flame brush with the wall in the oblique FWI depends on the flame angle and the value of $(x-{x}_{h})/h$ decreases with increasing flame angle. The flame angle increases with an increase in volume-integrated burning rate (Ghai et al. 2023b). The normalised volume-integrated burning rate $\Lambda ={\int }_{V}|{\dot{\omega }}_{F}|dV/[{\rho }_{0}{S}_{L}{h}^{2}\left({Y}_{Fu}-{Y}_{Fb}\right)]$ for the cases considered are shown in Table 1, which shows that $\Lambda$ assumes the highest value in case C and the lowest value in case A among the cases considered here.

**Table 1:** The numerical values of normalised volume-integrated burning rate ${\varvec{\Lambda}}={\int }_{{\varvec{V}}}|{\dot{{\varvec{\omega}}}}_{{\varvec{F}}}|{\varvec{d}}{\varvec{V}}/[{{\varvec{\rho}}}_{0}{{\varvec{S}}}_{{\varvec{L}}}{{\varvec{h}}}^{2}\left({{\varvec{Y}}}_{{\varvec{F}}{\varvec{u}}}-{{\varvec{Y}}}_{{\varvec{F}}{\varvec{b}}}\right)]$, for the cases under investigation in this study

$Case A$	$Case B$	$Case C$
$\Lambda =1.33$	$\Lambda =1.36$	$\Lambda =1.39$

The highest and the lowest values of $\Lambda$ in cases C and A, respectively can be explained in the following manner. For the case involving adiabatic walls (i.e. case B) the chemical reaction continues to the wall surface, whereas in the case of isothermal walls (i.e. Cases A and C) flame quenching occurs as a consequence to heat loss to the wall. In case C, the walls are maintained at a higher temperature, when compared to the reactant temperature throughout the domain, so a thermal boundary layer starts to develop from the inlet. Whereas in case A the thermal boundary layer develops in when the flame comes into the vicinity of the wall. The increase in temperature within the thermal boundary layer in case C even when the flame remains away serves to preheat the unburned gas mixture, which increases the burning rate within the thermal boundary layer. This is reflected in the higher values of $\Lambda$ in case C than in cases A and B. The augmented burning rate within the turbulent boundary layer gives rise to a reduction of the quenching distance in case C than in case A, which can be substantiated from Fig. 2b where the minimum Peclet number $P{e}_{min}$ (evaluated based on the wall-normal distance of ${\theta }^{*}=0.75$ isosurface because the peak heat release rate in the unstretched laminar flame occurs at ${\theta }^{*}\approx 0.75$ for the present thermochemistry) is smaller in case C than in case A. Figure 2b shows the maximum magnitudes of wall heat flux ${\Phi }_{w,max}$ and the minimum values of Peclet number $P{e}_{min}$ for turbulent V-flame, 2D laminar V-flame with the same centreline velocity as that of the turbulent case and 1D head-on quenching of laminar flame. It can be seen from Fig. 2b that the decrease in $P{e}_{min}$ due to the elevated wall temperature is valid not only for turbulent conditions but also for canonical laminar head-on quenching and laminar oblique quenching cases. The wall heat flux magnitude can be scaled as: ${|q}_{w}|\sim \lambda \Delta T/{\delta }_{Q}$ where $\Delta T$ is the temperature difference between the burned gas and the wall. This suggests that ${\Phi }_{w,max}$ scales as: ${\Phi }_{w,max}\sim \Delta T/[\left({T}_{ad}-{T}_{u}\right)P{e}_{min}]$. The value of ${\Phi }_{w,max}$ in Fig. 2b suggests that $P{e}_{min}$ changes in such a manner in the elevated wall temperature case that the decrease in $P{e}_{min}$ supersedes the drop in $\Delta T/\left({T}_{ad}-{T}_{u}\right)$ to give rise to an increase in ${\Phi }_{w,max}$. The wall-induced shear modifies the values of ${\Phi }_{w,max}$ and $P{e}_{min}$ in the 2D V-shaped laminar flame in comparison to the 1D planar laminar flame head-on quenching case for ${T}_{w}={T}_{u}$ and accordingly slightly greater values of $P{e}_{min}$ are obtained for the 2D V-shaped laminar flame than the 1D planar laminar flame head-on quenching case. Accordingly, ${\Phi }_{w,max}$ assumes lower values in the 2D V-shaped laminar flame than in the 1D planar laminar flame head-on quenching case where ${\theta }_{w}=0$. In the turbulent V-flame case with ${T}_{w}={T}_{u}$ (i.e., ${\theta }_{w}=0$), a greater rate of wall heat transfer caused by turbulent mixing leads to the flame quenching farther from the wall when compared to the 2D laminar V-flame with the same centreline velocity as that of the turbulent case. However, $P{e}_{min}$ in the 2D laminar V-flame is found to be significantly smaller than that the corresponding 1D head-on quenching value for the elevated wall temperature case (i.e., ${\theta }_{w}=0.3$). The upstream movement of the flame in both 2D laminar and 3D turbulent cases acts to reduce $P{e}_{min}$ in comparison to the corresponding 1D head-on quenching value for the elevated wall temperature case (i.e., ${\theta }_{w}=0.3$). Accordingly, ${\Phi }_{w,max}$ values in 2D laminar and 3D turbulent V-flame cases are greater than the corresponding 1D head-on quenching value for ${\theta }_{w}=0.3$. The combination of greater $\Delta T/\left({T}_{ad}-{T}_{u}\right)$ and smaller $P{e}_{min}$ gives rise to a significantly higher value of ${\Phi }_{w,max}$ in the laminar 2D laminar V-flame than in the 3D turbulent case for ${\theta }_{w}=0.3$.

It is worth noting that a previous experimental analysis (Kosaka et al. 2018) reported a decrease in the quenching distance and an increase in the maximum wall heat flux magnitude as a result of an increase in wall temperature in the case of interaction of V-shaped turbulent premixed flames with isothermal walls. The findings presented in Fig. 2 in the current analysis are in qualitative agreement with the experimental observations by Kosaka et al. ($2018$).

**Fig. 3:**
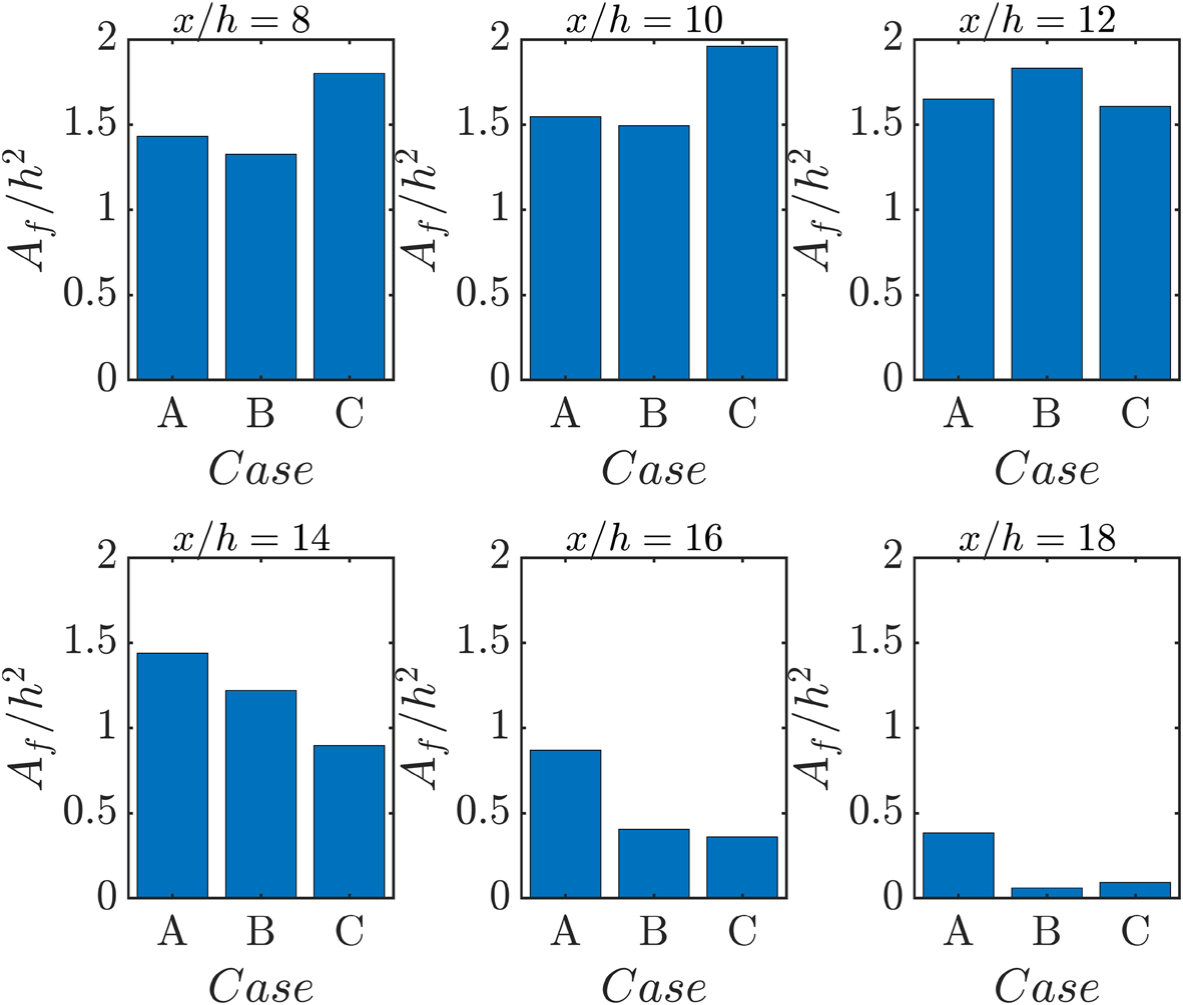
Variations of normalised turbulent flame surface area ${A}_{f}/{h}^{2}$ for flame–wall interaction of V-shaped premixed flames for cases A-C at $x/h= \text{8,10}, 12, \text{14,16}$ and $18$

**Fig. 4:**
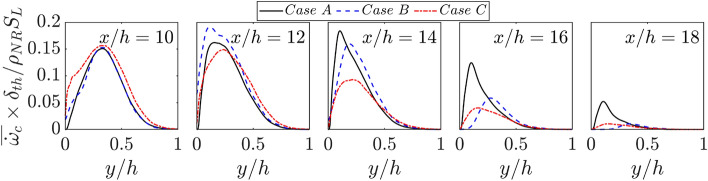
Variations of $\overline{\dot{{\omega }_{c}}}\times {\delta }_{th}/{{\rho }_{NR}S}_{L}$ with $y/h$ at at $x/h= 10, 12, \text{14,16}$ and $18$ for cases A-C

The augmented burning rate due to the preheating of reactants within the thermal boundary layer increases the local flame speed which can exceed the oncoming flow velocity in the near wall region leading to upstream movement of the flame in case C (see Fig. 1b). The same tendency can be seen for the adiabatic wall case (i.e., in case B) but this tendency in case B is much weaker than that in case C. This has implications for flame wrinkling in the cases considered here. The flame wrinkling can be expressed in terms of the ratio of the turbulent flame surface area ${A}_{f}={\int }_{V}\left|\nabla c\right|dV$ to the channel half height squared (i.e., ${h}^{2}$). It can be seen from Fig. 3 that ${A}_{f}/{h}^{2}$ assumes the highest value in case C and the lowest value in case B before the flame starts to interact with the wall (e.g., $x/h\le 10$). By contrast, just the opposite trend is observed for $x/h=12$. It is worth noting that $\left|\nabla c\right|$ drops as a result of FWI irrespective of the wall boundary condition (Ahmed et al. 2021b) and thus ${A}_{f}/{h}^{2}$ decreases with increasing $x/h$. Thus, Fig. 3 shows that a smaller value of ${A}_{f}/{h}^{2}$ for case C than in case A for $x/h\ge 12$, which shows that case C remains at a more advanced stage of quenching than in case A. This can further be substantiated by the distribution of $\overline{\dot{{\omega  }_{c}}}\times {\delta }_{th}/{\rho }_{NR}{S}_{L}$ with the normalised wall normal distance $y/h$ at different streamwise locations in Fig. 4, which shows that the highest (smallest) peak value of $\overline{\dot{{\omega  }_{c}}}\times {\delta }_{th}/{\rho }_{NR}{S}_{L}$ is obtained for case A (case C) for $x/h>12$.

### Wall Boundary Condition Effects on Scalar Statistics

**Fig. 5:**
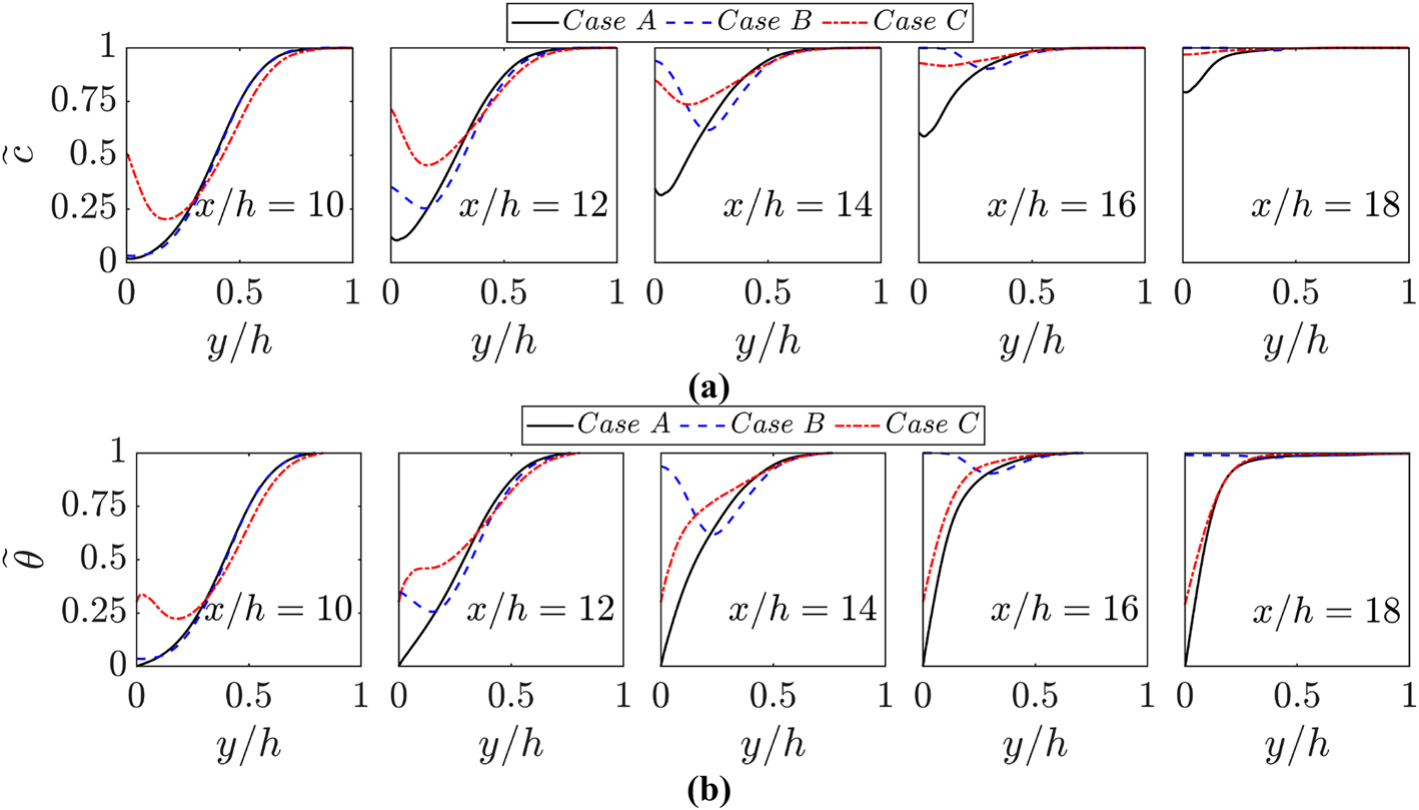
Variations of **a** $\widetilde{c}$ and **b** $\widetilde{\theta }$ with $y/h$ at $x/h= 10, 12, \text{14,16}$ and $18$ for cases A-C

The distributions of $\widetilde{c}$ and $\widetilde{\theta }$ with the normalised wall normal distance $y/h$ at different streamwise locations for all cases are shown in Fig. 5a, b, respectively. A comparison between Fig. 5a, b reveals that $\widetilde{c}=\widetilde{\theta }$ is maintained throughout the FWI in the case of adiabatic walls (i.e., case B), which is expected for low Mach number adiabatic conditions for unity Lewis number. However, the inequality between $\widetilde{c}$ and $\widetilde{\theta }$ in the near wall region can be seen for cases A and C. This difference between $\widetilde{c}$ and $\widetilde{\theta }$ in the case of isothermal boundary condition originates due to the differences in boundary conditions for the reaction progress variable (e.g., Neumann boundary condition) and non-dimensional temperature (e.g., Dirichlet boundary condition). The value of ($\widetilde{c}-\widetilde{\theta })$ provides a measure of non-adiabaticity in the case of FWI and $\widetilde{c}$ increases at the wall with the progress of flame quenching, as a consequence of the combination of the diffusion of unburned reactants from the quenched region into the burned gas and the diffusion of burned gas into the quenched zone. This behaviour is consistent with several previous findings (Ahmed et al. 2021a, b, c, 2023; Alshalaan and Rutland 1998, 2002; Bruneaux et al. 1996, 1997; Gruber et al. 2010; Lai and Chakraborty 2016a; Jiang et al. 2019; Ojo et al. 2021, 2022; Zentgraf et al. 2024). The values of $\widetilde{\theta }$ at the wall remain 0 and 0.3 for cases A and C, respectively due to the Dirichlet boundary condition. However, $\widetilde{\theta }$ at the wall increases as $\widetilde{c}$ increases in case B because the flame does not quench for adiabatic walls and the combustion process eventually stops when the reactants are fully consumed. Moreover, the profile of $\widetilde{\theta }$ for case C is considerably different to that of cases A and B where the flame remains away from the wall (i.e., $x/h\le 12$). This is a consequence of the fact that a thermal boundary layer develops upstream of the FWI event in case C because the wall temperature is different from the unburned reactant temperature in this case.

The $\widetilde{c}$ profiles close to the wall show a dip for all cases, which is more prominent in cases C and B (decreasing order) than in case A. This is a consequence of the curved contours of $\widetilde{c}$ due to the upstream movement of the flame close to the wall (see Fig. 1b). It can further be explained by expressing $\partial \widetilde{c}/\partial y$ in the following manner:

6 $$ \frac{{\partial \tilde{c}}}{{\partial y}} = \frac{1}{{\bar{\rho }}}\left[ {\overline{{\left( {\frac{{\partial \rho }}{{\partial y}}} \right)c}}  + \overline{{\left( {\frac{{\partial c}}{{\partial y}}} \right)\rho }} } \right] - \frac{{\tilde{c}}}{{\bar{\rho }}}\frac{{\partial \bar{\rho }}}{{\partial y}} $$

The first term on the right-hand side of Eq. 6 is negative but the other terms are positive during FWI, but the second term on the right-hand side is identically zero at the wall in the current configuration. Thus, a negative value of $\partial \widetilde{c}/\partial y$ at the wall is observed, as the magnitude of the first term overcomes that of the third term on the right-hand side of Eq. 6 because of a negative correlation between $\partial \rho /\partial y$ and $c$. The positive contribution of $(1/\overline{\rho })\left[\overline{\left(\partial c/\partial y\right)\rho }\right]$ away from the wall eventually yields positive $\partial \widetilde{c}/\partial y$ values. Because of $\widetilde{c}=\widetilde{\theta }$ in the adiabatic wall case (i.e., case B), the behaviour of $\partial \widetilde{\theta }/\partial y$ is qualitatively similar to that of $\partial \widetilde{c}/\partial y$ in case B and a negative value of $\partial \widetilde{\theta }/\partial y$ is obtained close to the wall in case B, whereas $\partial \widetilde{\theta }/\partial y$ assumes mostly positive values during the FWI in cases A and C.

**Fig. 6:**
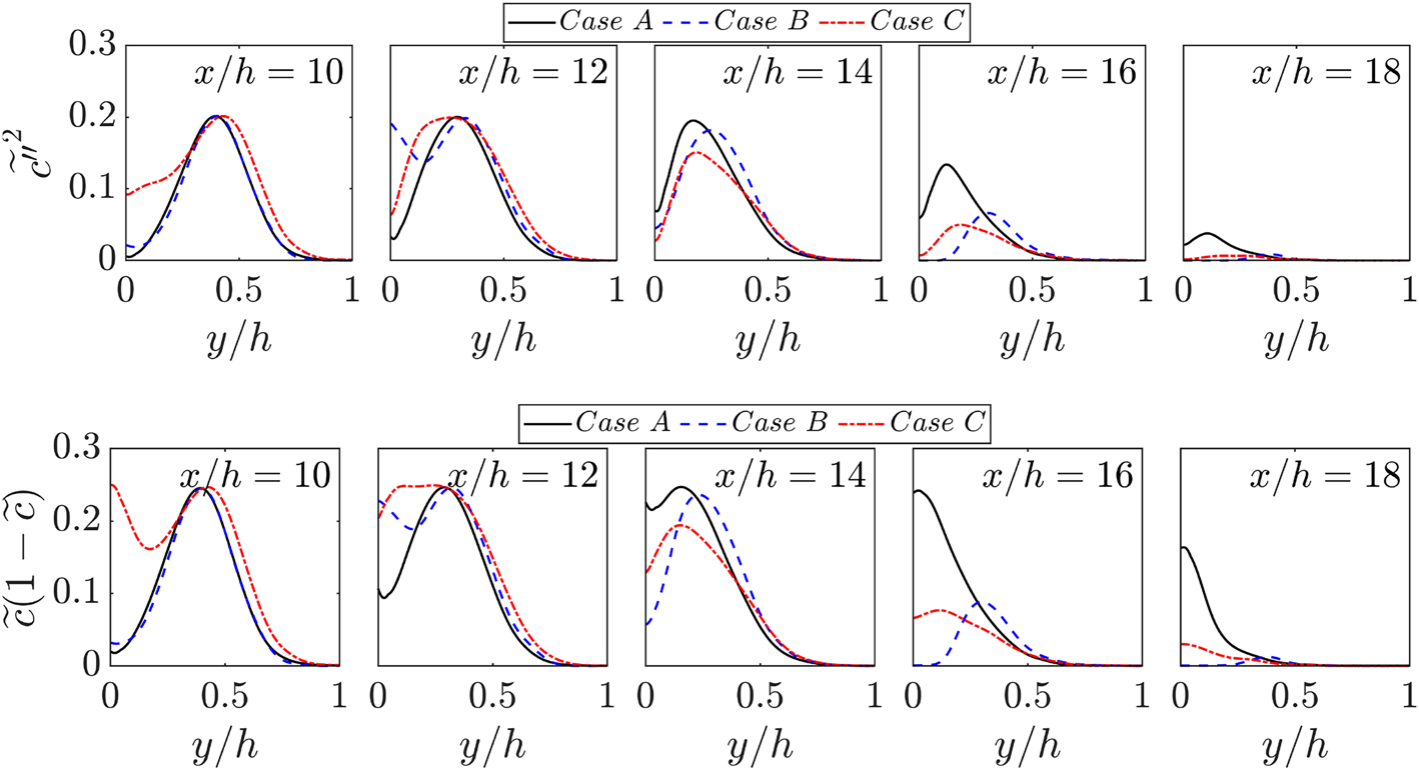
Variations of $\widetilde{{c}^{{\prime}{\prime}2}}$ and $\widetilde{c}(1-\widetilde{c})$ with $y/h$ at $x/h= 10, 12, \text{14,16}$ and $18$ for cases A-C

**Fig. 7:**
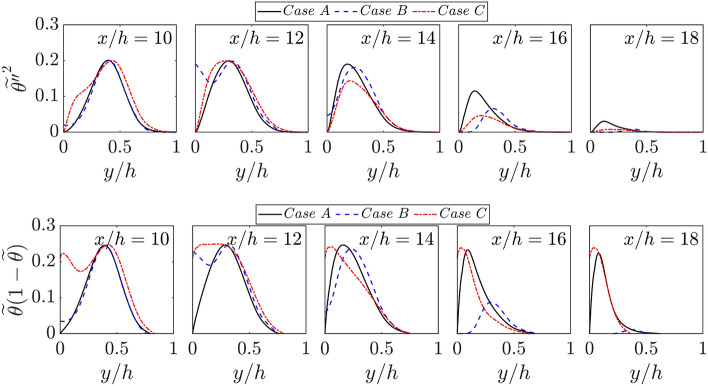
Variations of $\widetilde{{\theta }^{{\prime}{\prime}2}}$ and $\widetilde{\theta }(1-\widetilde{\theta })$ with $y/h$ at $x/h= 10, 12, \text{14,16}$ and $18$ for cases A-C

As the scalar variances $\widetilde{{c}^{{\prime}{\prime}2}}$ and $\widetilde{{\theta }^{{\prime}{\prime}2}}$ are needed for the purpose of modelling premixed combustion (Chakraborty and Swaminathan 2010; Lai and Chakraborty 2016c; Ghai et al. 2024), it is useful to analyse the influence of wall temperature on scalar variances. The variations of $\widetilde{{c}^{{\prime}{\prime}2}}$ and $\widetilde{{\theta }^{{\prime}{\prime}2}}$ with the normalised wall normal distance $y/h$ at different streamwise locations for all cases are shown in Figs. 6 and 7, respectively. Because of the perfect equality of $c$ and $\theta$ in the case of adiabatic walls, the scalar variances $\widetilde{{c}^{{\prime}{\prime}2}}$ and $\widetilde{{\theta }^{{\prime}{\prime}2}}$ are identical to each other at all stages of FWI in case B. However, the inequality of $\widetilde{{c}^{{\prime}{\prime}2}}$ and $\widetilde{{\theta }^{{\prime}{\prime}2}}$ is obtained in cases A and C in the near wall region. In cases A and C, $\widetilde{{\theta }^{{\prime}{\prime}2}}$ is identically zero at the wall due to the isothermal wall boundary condition. However, $\widetilde{{c}^{{\prime}{\prime}2}}$ assumes non-zero values at the wall during the early stages of FWI. Figure 6 shows that $\widetilde{{c}^{{\prime}{\prime}2}}$ remain smaller than $\widetilde{c}(1-\widetilde{c})$ throughout the duration of FWI. For a bimodal distribution of the probability density function of $c$, one obtains $\widetilde{{c}^{{\prime}{\prime}2}}=\widetilde{c}(1-\widetilde{c})$ (Bray et al. 1985). The deviation of $\widetilde{{c}^{{\prime}{\prime}2}}$ from $\widetilde{c}(1-\widetilde{c})$ provides the measure of the departure of the probability density function of $c$ from a bimodal distribution (Ahmed et al. 2021c; Chakraborty and Swaminathan 2010; Lai and Chakraborty 2016c; Ghai et al. 2024), and this aspect is particularly prevalent in the vicinity of the wall for cases A and C. A similar qualitative behaviour is observed for both $\widetilde{{\theta }^{{\prime}{\prime}2}}$ and $\widetilde{\theta }(1-\widetilde{\theta })$ (i.e., $\widetilde{{\theta }^{{\prime}{\prime}2}}<\widetilde{\theta }(1-\widetilde{\theta })$) for case C. Although $\widetilde{{\theta }^{{\prime}{\prime}2}}<\widetilde{\theta }(1-\widetilde{\theta })$ is mostly obtained for case A but both $\widetilde{{\theta }^{{\prime}{\prime}2}}$ and $\widetilde{\theta }(1-\widetilde{\theta })$ assume zero values at the wall in this case. In all cases, both $\widetilde{{c}^{{\prime}{\prime}2}}$ and $\widetilde{{\theta }^{{\prime}{\prime}2}}$ decrease with the progress of FWI irrespective of wall boundary condition. This behaviour is explained and the closures of $\widetilde{{c}^{{\prime}{\prime}2}}$ and $\widetilde{{\theta }^{{\prime}{\prime}2}}$ have been discussed elsewhere (Lai and Chakraborty 2016c; Ghai et al. 2024) by the authors and thus this is not explained here. Interested readers are referred to Lai Lai and Chakraborty (2016c) and Ghai et al. (2024) for further information in this regard.

**Fig. 8:**
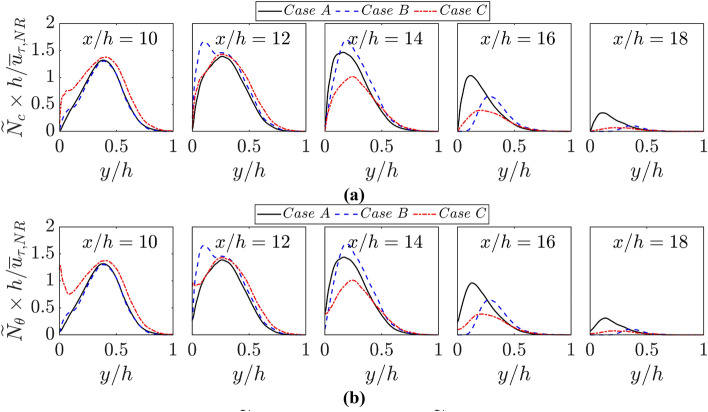
Variations of **a** ${\widetilde{N}}_{c}\times h/{\overline{u} }_{\tau ,NR}$ and **b** ${\widetilde{N}}_{\theta }\times h/{\overline{u} }_{\tau ,NR}$ with $y/h$ at $x/h= 10, 12, \text{14,16}$ and $18$ for cases A-C

The closures of $\widetilde{{c}^{{\prime}{\prime}2}}$ and $\widetilde{{\theta }^{{\prime}{\prime}2}}$ need the statistical information of their Favre-averaged scalar dissipation rates ${\widetilde{N}}_{c}=\overline{\rho D\nabla c\bullet \nabla c }/\overline{\rho }$ and ${\widetilde{N}}_{\theta }=\overline{\rho {\alpha  }_{T}\nabla \theta \bullet \nabla \theta }/\overline{\rho }$, respectively. As all cases have a unity Lewis number for the current analysis, the relative magnitudes of ${\widetilde{N}}_{c}$ and ${\widetilde{N}}_{\theta }$ are indicative of the magnitudes of $|\nabla c|$ and $|\nabla \theta |$. The variations of ${\widetilde{N}}_{c}\times h/{\overline{u} }_{\tau ,NR}$ and ${\widetilde{N}}_{\theta }\times h/{\overline{u} }_{\tau ,NR}$ with the normalised wall normal distance $y/h$ at different streamwise locations for all cases are shown in Fig. 8a, b, respectively. A comparison between Fig. 8a, b reveals that ${\widetilde{N}}_{c}$ and ${\widetilde{N}}_{\theta }$ are identical for the adiabatic wall case (i.e., case B) because of $c=\theta$. Both ${\widetilde{N}}_{c}$ and ${\widetilde{N}}_{\theta }$ assume similar peak values within the reacting flow turbulent boundary layers for all cases. The differences between ${\widetilde{N}}_{c}$ and ${\widetilde{N}}_{\theta }$ for cases A and C arise only in the near wall region and ${\widetilde{N}}_{c}<{\widetilde{N}}_{\theta }$ is obtained in this region because $\partial c/\partial y$ vanishes at the wall but $\partial \theta /\partial y$ assumes significant non-zero values at the wall within the thermal boundary layer during FWI in the case of isothermal boundary condition. Figure 8a, b also show that both ${\widetilde{N}}_{c}$ and ${\widetilde{N}}_{\theta }$ drop in magnitude with the progress of FWI, which is consistent with previous findings (Ahmed et al. 2021b; Kai et al. 2023) and interested readers are directed to Ahmed et al. (2021b) and Kai et al. (2023) for the detailed physical explanations for this behaviour.

**Fig. 9:**
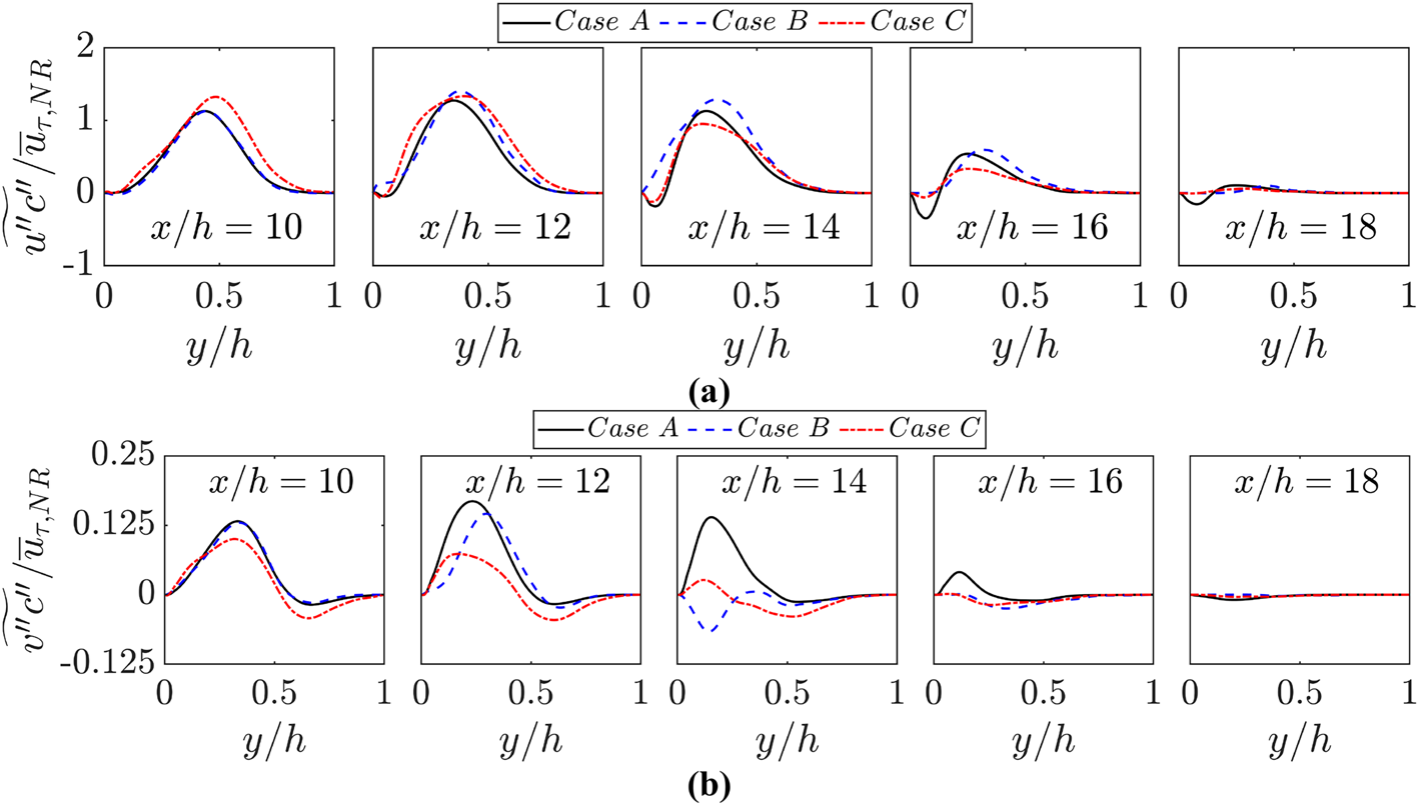
Variations of **a** $\widetilde{{u^{{\prime}{\prime}}c}^{{\prime}{\prime}}}/{\overline{u} }_{\tau ,NR}$ and $\widetilde{{v^{{\prime}{\prime}}c}^{{\prime}{\prime}}}/{\overline{u} }_{\tau ,NR}$ with $y/h$ at $x/h= 10, 12, \text{14,16}$ and $18$ for cases A-C

It can be appreciated from Eqs. 2 and 3 that the statistical behaviour of turbulent scalar fluxes $\widetilde{{u^{{\prime}{\prime}}c}^{{\prime}{\prime}}}=\overline{\rho {u}_{j}^{{\prime}{\prime}}{{{c}^{{\prime}{\prime}}}}}/\overline{\rho }$ and $\widetilde{{{u}^{{\prime}{\prime}}\theta }^{{\prime}{\prime}}}=\overline{\rho {u}_{j}^{{\prime}{\prime}}{{{\theta }^{{\prime}{\prime}}}}}/\overline{\rho }$ are needed for the closure of $\widetilde{c}, \widetilde{{c}^{{\prime}{\prime}2}}$ and $\widetilde{\theta }, \widetilde{{\theta }^{{\prime}{\prime}2}}$ transport equations. The variations of $\widetilde{{u^{{\prime}{\prime}}c}^{{\prime}{\prime}}}/{\overline{u} }_{\tau ,NR}$ and $\widetilde{{v^{{\prime}{\prime}}c}^{{\prime}{\prime}}}/{\overline{u} }_{\tau ,NR}$ (where $u={u}_{1}$; $v={u}_{2}$; $w={u}_{3}$ and $x={x}_{1}$; $y={x}_{2}$; $z={x}_{3}$) with the normalised wall normal distance $y/h$ at different streamwise locations for all cases are shown in Fig. 9a, b, respectively. The qualitative and quantitative behaviours of $\widetilde{{{u}^{{\prime}{\prime}}\theta }^{{\prime}{\prime}}}$ and $\widetilde{{{v}^{{\prime}{\prime}}\theta }^{{\prime}{\prime}}}$ are similar to those of $\widetilde{{u^{{\prime}{\prime}}c}^{{\prime}{\prime}}}$ and $\widetilde{{v^{{\prime}{\prime}}c}^{{\prime}{\prime}}}$, respectively and thus are not explicitly shown in this paper. Figure 9a shows that the wall boundary condition does not significantly influence the behaviour of turbulent scalar flux in the streamwise direction $\widetilde{{u^{{\prime}{\prime}}c}^{{\prime}{\prime}}}$, which assumes negative values close to the wall and positive values away from the wall for cases A and C. In case B, $\widetilde{{u^{{\prime}{\prime}}c}^{{\prime}{\prime}}}$ assumes positive values throughout the reacting flow boundary layer. It can be seen from Fig. 9b that $\widetilde{{v^{{\prime}{\prime}}c}^{{\prime}{\prime}}}$ assumes positive values close to the wall when the flame approaches the wall during the early stages of FWI, but negative values are obtained in the burned gas away from the wall. With the progress of FWI, $\widetilde{{v^{{\prime}{\prime}}c}^{{\prime}{\prime}}}$ assumes negative values within the reacting flow boundary layer and this trend is particularly more prominent for the case with adiabatic walls (i.e., case B) than in cases A and C. The magnitudes of both $\widetilde{{u^{{\prime}{\prime}}c}^{{\prime}{\prime}}}$ and $\widetilde{{v^{{\prime}{\prime}}c}^{{\prime}{\prime}}}$ decrease with the progress of FWI and thus their magnitudes decrease with increasing $x/h$.

**Fig. 10:**
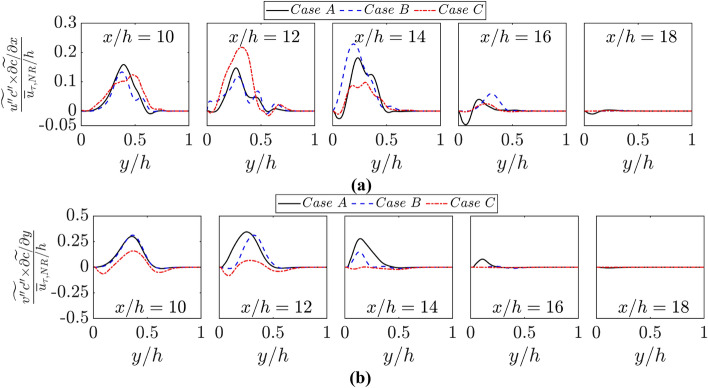
Variations of **a** $\widetilde{{u^{{\prime}{\prime}}c}^{{\prime}{\prime}}}\times \partial \widetilde{c}/\partial x\times h/{\overline{u} }_{\tau ,NR}$ and **b** $\widetilde{{v^{{\prime}{\prime}}c}^{{\prime}{\prime}}}\times \partial \widetilde{c}/\partial y\times h/{\overline{u} }_{\tau ,NR}$ with $y/h$ at $x/h= 10, 12, \text{14,16}$ and $18$ for cases A-C

It is important to consider the values of $\widetilde{{u^{{\prime}{\prime}}c}^{{\prime}{\prime}}}\times \partial \widetilde{c}/\partial x$ and $\widetilde{{v^{{\prime}{\prime}}c}^{{\prime}{\prime}}}\times \partial \widetilde{c}/\partial y$ because they assume positive (negative) values for the counter-gradient (gradient) type of transport. The variations of $\widetilde{{u^{{\prime}{\prime}}c}^{{\prime}{\prime}}}\times \partial \widetilde{c}/\partial x\times h/{\overline{u} }_{\tau ,NR}$ and $\widetilde{{v^{{\prime}{\prime}}c}^{{\prime}{\prime}}}\times \partial \widetilde{c}/\partial y\times h/{\overline{u} }_{\tau ,NR}$ with $y/h$ at different streamwise locations for all cases are shown in Fig. 10. It can be seen from Fig. 10 that predominantly counter-gradient behaviour is obtained for both $\widetilde{{u^{{\prime}{\prime}}c}^{{\prime}{\prime}}}$ and $\widetilde{{v^{{\prime}{\prime}}c}^{{\prime}{\prime}}}$ in cases A-C in the regions where the influence of chemical reaction is strong, as the velocity jump due to thermal expansion effects overwhelms the transport due to turbulent velocity fluctuations (Veynante et al. 1997). However, the gradient type of transport (i.e., $\widetilde{{u^{{\prime}{\prime}}c}^{{\prime}{\prime}}}\times \partial \widetilde{c}/\partial x<0$ and $\widetilde{{v^{{\prime}{\prime}}c}^{{\prime}{\prime}}}\times \partial \widetilde{c}/\partial y<0$) is obtained in the zones where the flame is quenched as a result of wall heat loss in cases A and C, and also towards the burned gas side of the flame brush where the effects of thermal expansion are weak. However, for case C counter-gradient transport of $\widetilde{{v^{{\prime}{\prime}}c}^{{\prime}{\prime}}}$ close to the wall where the turbulent velocity fluctuations in the wall normal direction remain small but a gradient type of transport is observed for $\widetilde{{v^{{\prime}{\prime}}c}^{{\prime}{\prime}}}$ away from the wall. However, the behaviour of $\widetilde{{u^{{\prime}{\prime}}c}^{{\prime}{\prime}}}\times \partial \widetilde{c}/\partial x$ and $\widetilde{{v^{{\prime}{\prime}}c}^{{\prime}{\prime}}}\times \partial \widetilde{c}/\partial y$ in case B is found to be qualitatively similar to the trends observed for cases A and C away from the wall. Moreover, $\widetilde{{v^{{\prime}{\prime}}c}^{{\prime}{\prime}}}\times \partial \widetilde{c}/\partial y$ in case C is found to be qualitatively different to the trends observed for cases A and B at $x/h=10, 12$ and 14. The differences in behaviours of $\widetilde{{v{\prime}{\prime}c}^{{\prime}{\prime}}}$ and $\widetilde{{v^{{\prime}{\prime}}c}^{{\prime}{\prime}}}\times \partial \widetilde{c}/\partial y$ between case C and cases A and B originate due to the differences in turbulence statistics. These effects are primarily felt in the turbulent scalar flux in the wall normal direction and the streamwise component remains mostly unaltered because the flame normal acceleration effects are mostly felt in the $y-$ direction due to the small angle of flame intersection with the wall surface. In order to explain the scalar flux behaviours further, it will be instructive to analyse the wall boundary condition effects on the turbulent flow statistics, which are addressed in the next sub-section.

### Wall Boundary Condition Effects on Flow Statistics

**Fig. 11:**
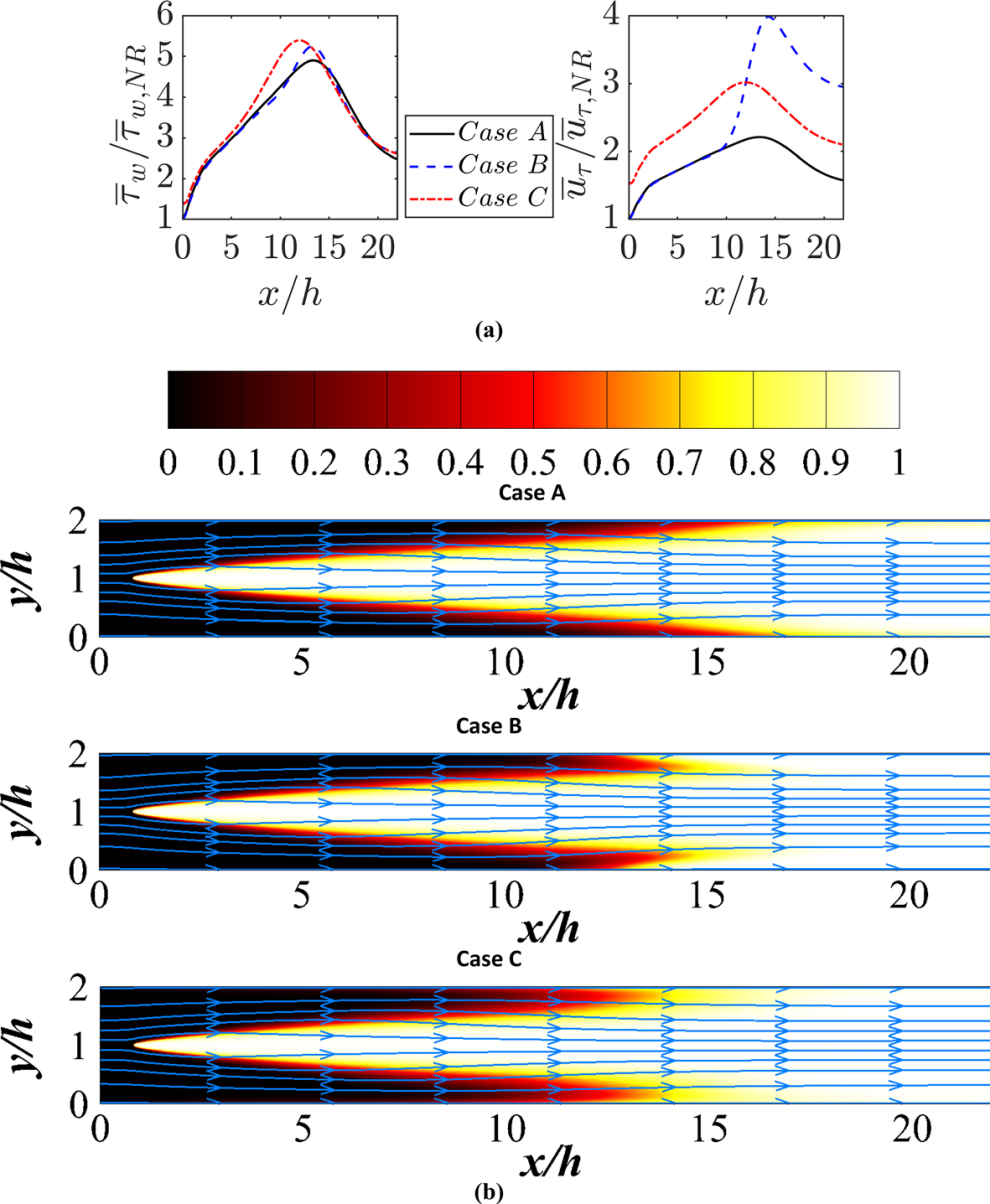
**a** Variations of ${\overline{\tau }}_{w}/{\overline{\tau }}_{w,NR}$ and ${\overline{u} }_{\tau }/{\overline{u} }_{\tau ,NR}$ with $x/h$ for cases A-C; **b** mean velocity-based streamlines superimposed on $\widetilde{c}$ field in the V-flame configuration

The variations of normalised mean wall shear stress ${\overline{\tau }}_{w}/{\overline{\tau }}_{w,NR}$ and normalised friction velocity ${\overline{u} }_{\tau }/{\overline{u} }_{\tau ,NR}$ (where ${\overline{u} }_{\tau }=\sqrt{\left|{\overline{\tau }}_{w}\right|/{\overline{\rho }}_{w}}$) with the normalised streamwise distance $x/h$ are shown in Fig. 11a. It can be seen from Fig. 11a that ${\overline{\tau }}_{w}$ increases from ${\overline{\tau }}_{w,NR}$ at the inlet before FWI takes place. This is a consequence of the flow deviation towards the wall due to the flame holder as can be seen from the mean velocity-based streamline patterns shown in Fig. 11b. This is consistent with previous findings by Ahmed et al. (2023). The highest value of ${\overline{\tau }}_{w}/{\overline{\tau }}_{w,NR}$ is obtained for case C because the velocity gradient is enhanced by the thermal expansion as a result of elevated wall temperature. The momentum redistribution caused by thermal expansion occurs in the wall normal direction due to thermal expansion during FWI, which can be seen from the movement of streamlines away from the wall in the vicinity of wall boundaries in Fig. 11b. This gives rise to a reduction of the magnitude of the wall normal gradient of the streamwise velocity leading to a drop in ${\overline{\tau }}_{w}$ with the progress of FWI.

During FWI, the mean friction velocity ${\overline{u} }_{\tau }$ values begin to diminish in both instances involving isothermal walls. In contrast, for the adiabatic wall condition, the mean friction velocity values initially rise, followed by a subsequent decrease, yet these values persist at a level surpassing those observed in both cases with isothermal wall condition. The values of ${\overline{u} }_{\tau }/{\overline{u} }_{\tau ,NR}$ remain almost similar to each other for cases A and B before FWI but ${\overline{\rho }}_{w}$ drops in comparison to ${\rho }_{NR}$ in the case of adiabatic wall due to thermal expansion as a result of chemical reactions at the wall surface and this leads to an increase in ${\overline{u} }_{\tau }/{\overline{u} }_{\tau ,NR}$ during FWI in case B in comparison to that obtained for case A where ${\overline{\rho }}_{w}\approx {\rho }_{R}$ is obtained. The ${\overline{u} }_{\tau }/{\overline{u} }_{\tau ,NR}$ values for case C are found to be greater than those in case A although ${\overline{\tau }}_{w}/{\overline{\tau }}_{w,NR}$ values are mostly comparable for these two cases. The mean density at the wall is smaller in case C than in case A due to elevated wall temperature and this gives rise to greater values of ${\overline{u} }_{\tau }/{\overline{u} }_{\tau ,NR}$ in case C than in case A. This behaviour has implications for the variation of ${u}^{+}=\widetilde{u}/{\overline{u} }_{\tau }$ with ${y}^{+}={\overline{u} }_{\tau }y/{\nu }_{w}$ (where ${\nu }_{w}$ is the kinematic viscosity at the wall) within the reacting flow turbulent boundary layers.

**Fig. 12:**
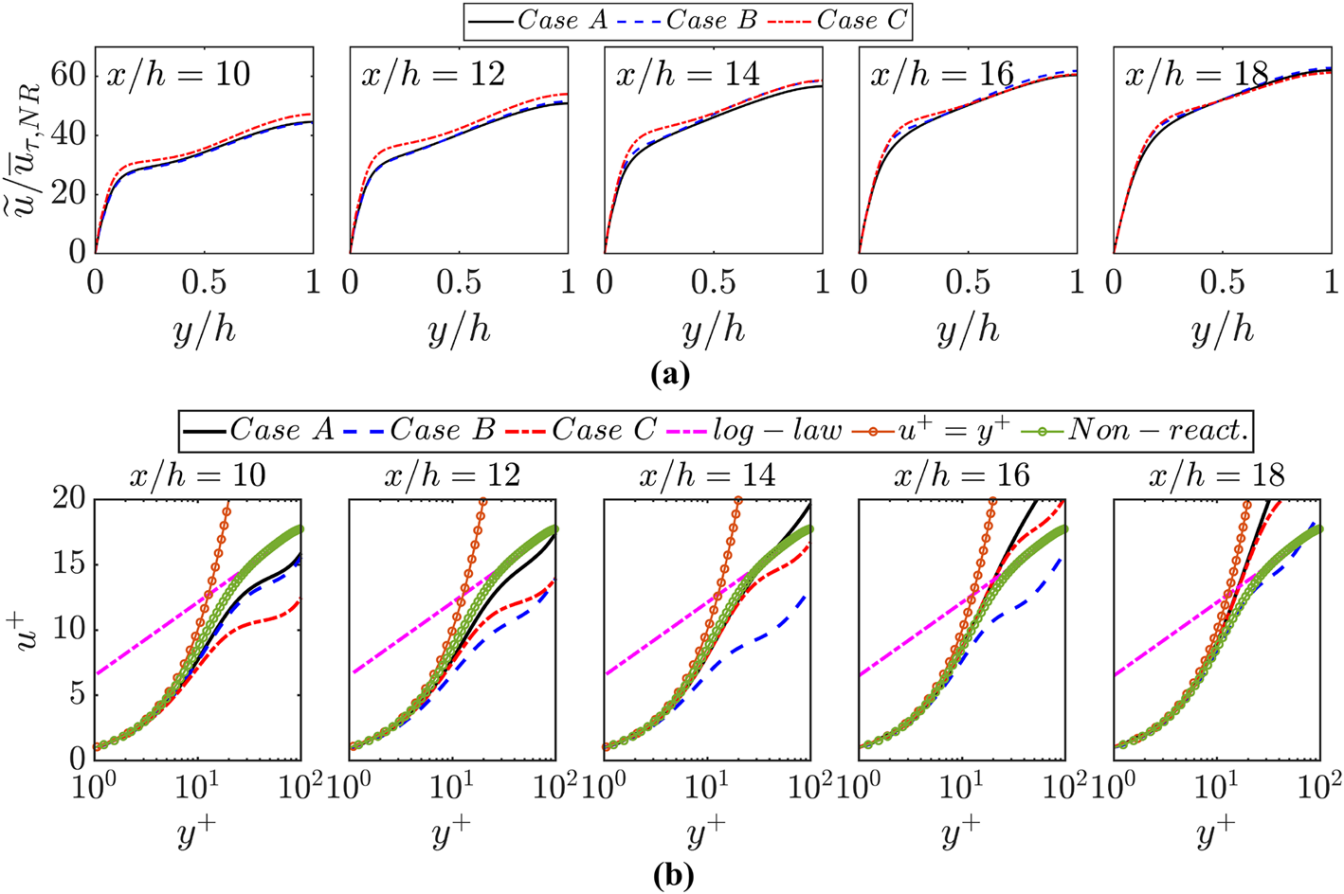
**a** Variations of $\widetilde{u}/{\overline{u} }_{\tau ,NR}$ with $y/h$ at $x/h= 10, 12, \text{14,16}$ and $18$ for cases A-C; **b** variations of ${u}^{+}$ with ${y}^{+}$ at $x/h= 10, 12, \text{14,16}$ and $18$ for cases A-C

The variations of $\widetilde{u}/{\overline{u} }_{\tau ,NR}$ with $y/h$ at different streamwise locations are shown in Fig. 12a where the corresponding variations of ${u}^{+}$ with ${y}^{+}$ are also shown. It can be seen from Fig. 12a that case C shows marginally higher value of $\widetilde{u}/{\overline{u} }_{\tau ,NR}$ than that in cases A and B at the beginning of FWI but $\widetilde{u}/{\overline{u} }_{\tau ,NR}$ values remain comparable for all the cases at the later stages of FWI. The thermal expansion effects due to the elevated wall temperature act to increase the velocity magnitude $\widetilde{u}/{\overline{u} }_{\tau ,NR}$ in case C in comparison to cases A and B. As the burned gas temperature is the same for all these cases, the magnitudes of $\widetilde{u}/{\overline{u} }_{\tau ,NR}$ remain comparable during FWI for cases A-C.

It is worth noting that different values of ${y}^{+}$ are obtained for a given value of $y/h$ at different values of $x/h$ for the cases considered here. The aforementioned differences in behaviour of ${\overline{u} }_{\tau }$ lead to differences in the variations of ${u}^{+}$ with ${y}^{+}$ between the cases considered here (see Fig. 12b). The usual relations for viscous sub-layer (i.e., ${u}^{+}={y}^{+}$) and log law (i.e., ${u}^{+}=\left(1/\kappa \right)log{y}^{+}+B$ with $\kappa =0.41$ being the von-Karman’s constant and $B$ is the appropriate constant for $R{e}_{\tau }=110$) are also shown in Fig. 12b along with the variations of ${u}^{+}$ with ${y}^{+}$ for the corresponding non-reacting flow with the unburned gas properties at $R{e}_{\tau }=110$. It can be seen from Fig. 12b that ${u}^{+}={y}^{+}$ and log-law predicts the variation of ${u}^{+}$ for ${y}^{+}<5$ and ${y}^{+}\ge 11.7$ for the non-reacting flow. In the reacting cases, a good agreement between DNS data and the prediction of ${u}^{+}={y}^{+}$ is obtained within the viscous sub-layer but the log-law does not remain valid for ${y}^{+}\ge 11.7$ during FWI for all the reacting flow cases irrespective of the thermal wall boundary condition. This is consistent with the findings of previous experimental (Zentgraf et al. 2024) and computational (Ahmed et al. 2021a, 2023, 2024; Ghai et al. 2023a) studies. It has been explained in detail elsewhere (Ahmed et al. 2024; Ghai et al. 2023a) that the underlying assumptions for the log-law are rendered invalid during FWI and these aspects are valid also for the database considered in the current analysis. At the early stages of FWI (e.g., $x/h=10$), the lowest value of ${u}^{+}$ is obtained for case C for a given value of ${y}^{+}$ because ${\overline{u} }_{\tau }$ is the highest for case C and $\widetilde{u}$ values are comparable for all cases. However, for $x/h\ge 12$, the highest (lowest) value of ${u}^{+}$ is obtained for case A (case B) for a given value of ${y}^{+}$ because the lowest (highest) value of ${\overline{u} }_{\tau }$ is obtained for case A and case B during FWI (see Fig. 11a). The value of ${u}^{+}$ for a given value of ${y}^{+}$ increases with increasing $x/h$ because of the combination of the drop in ${\overline{u} }_{\tau }$ with the progress of FWI and an increasing trend of $\widetilde{u}$ with $y$ within the boundary layer.

**Fig. 13:**
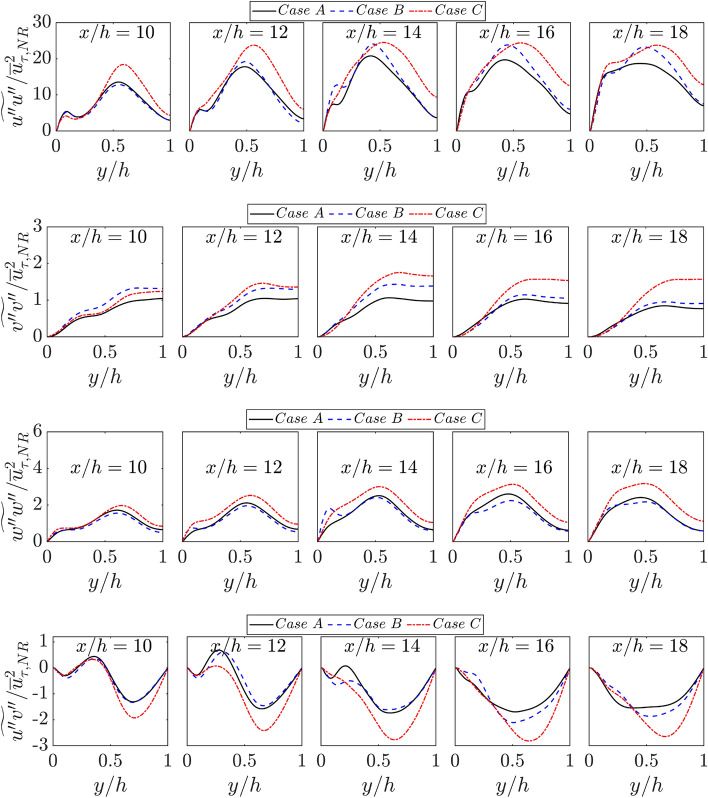
Variations of $\{ \widetilde{{u^{{\prime \prime }} u^{{''}} }},\widetilde{{v^{{\prime \prime }} v^{{''}} }},\widetilde{{w^{{\prime \prime }} w^{{''}} }}$ and $\widetilde{{u^{{\prime}{\prime}}v}^{{\prime}{\prime}}}\}/{\overline{u} }_{\tau ,NR}^{2}$ with $y/h$ at $x/h= 10, 12, \text{14,16}$ and $18$ for cases A-C

Finally, the variations of the Reynolds stress components (i.e., $\{\widetilde{{u^{{\prime}{\prime}}u}^{{\prime}{\prime}}},\widetilde{{v^{\prime}{^\prime}v}^{{\prime}{\prime}}},\widetilde{{w^{\prime}{^\prime}w}^{{\prime}{\prime}}}$ and $\widetilde{{u^{{\prime}{\prime}}v}^{{\prime}{\prime}}}\}/{\overline{u} }_{\tau ,NR}^{2}$ with the normalised wall normal distance $y/h$ for all cases are shown in Fig. 13. It can be seen from Fig. 13 that $\widetilde{{u^{{\prime}{\prime}}u}^{{\prime}{\prime}}}$ is the dominant component among the diagonal components of the Reynolds stress tensor for all cases and $\widetilde{{v^{{\prime}{\prime}}v}^{{\prime}{\prime}}}$ assumes the smallest magnitude among the diagonal elements in these cases. It can further be seen from Fig. 13 that the magnitudes of Reynolds stresses are the smallest in case A among the cases considered here because the thermal expansion effects in case A are weaker than in cases B and C.

The variation of $\widetilde{{u^{{\prime}{\prime}}v}^{{\prime}{\prime}}}/{\overline{u} }_{\tau ,NR}^{2}$ in the wall normal direction is also shown in Fig. 13. A positive value of $\widetilde{{u^{{\prime}{\prime}}v}^{{\prime}{\prime}}}$ is indicative of the invalidity of Boussinesq’s hypothesis, $\widetilde{{u^{{\prime}{\prime}}v}^{{\prime}{\prime}}}=-{\nu }_{t}(\partial \widetilde{u}/\partial y)$, as $\partial \widetilde{u}/\partial y$ remains positive in the region given by $0<y/h<1.0$ and can be seen in Fig. 12 (note that for the top wall (i.e., $1.0<y/h<2.0$), $y$ and $v$ should be treated as the wall normal distance and wall normal velocity). Thus, negative values of $\widetilde{{u^{{\prime}{\prime}}v}^{{\prime}{\prime}}}$ is consistent with the qualitative behaviour predicted by $\widetilde{{u^{{\prime}{\prime}}v}^{{\prime}{\prime}}}=-{\nu }_{t}(\partial \widetilde{u}/\partial y)$. According to Bray et al. (1985) $\widetilde{u^{{\prime}{\prime}}v{\prime}{\prime}}$ can be expressed as:

7 $$ \widetilde{{u^{{\prime \prime }} v^{{\prime \prime }} }} = \tilde{c}\left( {1 - \tilde{c}} \right)\left[ {\overline{{\left( u \right)}} _{P}  - \overline{{\left( u \right)}} _{R} } \right]\left[ {\overline{{\left( v \right)}} _{P}  - \overline{{\left( v \right)}} _{R} } \right] + ~\left( {1 - \tilde{c}} \right)\overline{{\left( {u^{\prime } v^{\prime } } \right)}} _{R}  + \tilde{c}\overline{{\left( {u^{\prime } v^{\prime } } \right)}} _{P}  + O\left( {\gamma _{c} } \right) $$

where ${\overline{\left(q\right)}}_{R}$ and ${\overline{\left(q\right)}}_{P}$ are the conditional mean values in unburned reactants and products of a general quantity $q$ and $O({\gamma }_{c})$ is the contribution of the burning mixture. The counter-gradient behaviour of $\widetilde{u^{{\prime}{\prime}}v^{{\prime}{\prime}}}$ occurs when the contribution due to velocity jump across the flame brush (i.e., which leads to positive values for the 1st term on the right-hand side of Eq. 1) dominates over the turbulent velocity fluctuation contributions (i.e., 2nd and 3rd terms on the right-hand side of Eq. 7) (Bray et al. 1985; Veynante et al. 1997; Chakraborty et al. 2011) but a gradient type of transport is obtained when fluid turbulence contributions overwhelm flame normal acceleration due to thermal expansion. During the early stages of flame quenching (e.g., at $x/h\le 12$) $\widetilde{u^{{\prime}{\prime}}v^{{\prime}{\prime}}}$ assumes negative values both in the unburned gas and burned gas regions in cases A, B and C where the effects of thermal expansion are not strong in the absence of chemical heat release. By contrast, positive values of $\widetilde{u^{{\prime}{\prime}}v^{{\prime}{\prime}}}$ are obtained in the regions of heat release during the early stages of FWI (i.e., $x/h\le 12$) in these cases. However, only the negative values of $\widetilde{u^{{\prime}{\prime}}v^{{\prime}{\prime}}}$ are obtained as FWI progresses because the effects of thermal expansion either weaken or vanish at the advanced stages of FWI due to flame quenching in cases A and C and due to consumption of the available fuel in case B. The magnitude of the negative value of $\widetilde{u^{{\prime}{\prime}}v^{{\prime}{\prime}}}$ is greater for case C than in case A.

The anisotropy associated with $\widetilde{{u}_{i}^{{\prime}{\prime}}{u}_{j}^{{\prime}{\prime}}}$ can be expressed as Lumley and Newman (1977), Antonia et al. (1994), Leschziner (2015):

8 $${b}_{ij}=\widetilde{{u}_{i}^{{\prime}{\prime}}{u}_{j}^{{\prime}{\prime}}}/2\widetilde{k}-{\delta }_{ij}/3$$

where $\widetilde{k}=\widetilde{{u}_{j}^{{\prime}{\prime}}{u}_{i}^{{\prime}{\prime}}}/2$ and $\widetilde{\varepsilon }=\overline{\mu (\partial {u}_{i}^{{\prime}{\prime}}/\partial {x}_{k})(\partial {u}_{i}^{{\prime}{\prime}}/\partial {x}_{k}})/\overline{\rho }={\widetilde{\varepsilon }}_{ii}/2$ are the turbulent kinetic energy and its dissipation rate, respectively. The first invariant of the above tensor is identically zero as it is trace-free (i.e., ${b}_{ii}={d}_{ii}=0$) but the second (i.e., $I{I}_{b}$) and third (i.e., $II{I}_{b}$) invariants are given by Lumley and Newman (1977), Antonia et al. (1994), Leschziner (2015):

9 $$ II_{b} = - b_{ij} b_{ji} /2;\;III_{b} = b_{ij} b_{jk} b_{ki} /3 $$

Based on the eigenvalues of the anisotropy tensors, the following limiting conditions of the realisable states of turbulence can be obtained:

*One component (1C) limit*: One of the eigenvalues of $\widetilde{{u}_{i}^{{\prime}{\prime}}{u}_{j}^{{\prime}{\prime}}}$ assumes non-zero values and the eigenvalues of the anisotropy tensor can be expressed as: $\left\{{\lambda }_{1},{\lambda }_{2},{\lambda }_{3}\right\}=\{2/3,-1/3,-1/3\}$.

*Two-component (2C) axisymmetric limit*: For this limit, two of the eigenvalues of $\widetilde{{u}_{i}^{{\prime}{\prime}}{u}_{j}^{{\prime}{\prime}}}$ are non-zero and the eigenvalues of the anisotropy tensor are given by $\left\{{\lambda }_{1},{\lambda }_{2},{\lambda }_{3}\right\}=\{1/6,1/6,-1/3\}$.

*Three-component (3C) isotropy limit*: In this limit the eigenvalues of $\widetilde{{u}_{i}^{{\prime}{\prime}}{u}_{j}^{{\prime}{\prime}}}$ are non-zero and equal, while the eigenvalues of the anisotropy tensor are $\left\{{\lambda }_{1},{\lambda }_{2},{\lambda }_{3}\right\}=\{\text{0,0},0\}$.

These limits yield the borders of the Lumley triangle in the following manner (Lumley and Newman 1977; Antonia et al. 1994; Leschziner 2015):

- 2 Component limit, which translates to pancake-like turbulence structures with ${\lambda }_{1}>{\lambda }_{2}$ and ${\lambda }_{3}$=0.
- Axisymmetric expansion where rod-like turbulence structures with ${\lambda }_{1}>{\lambda }_{2}{=\lambda }_{3}$ are realised.
- Axisymmetric compression where disc-like turbulence structures are obtained with ${\lambda }_{1}={\lambda }_{2}{>\lambda }_{3}$.

**Fig. 14:**
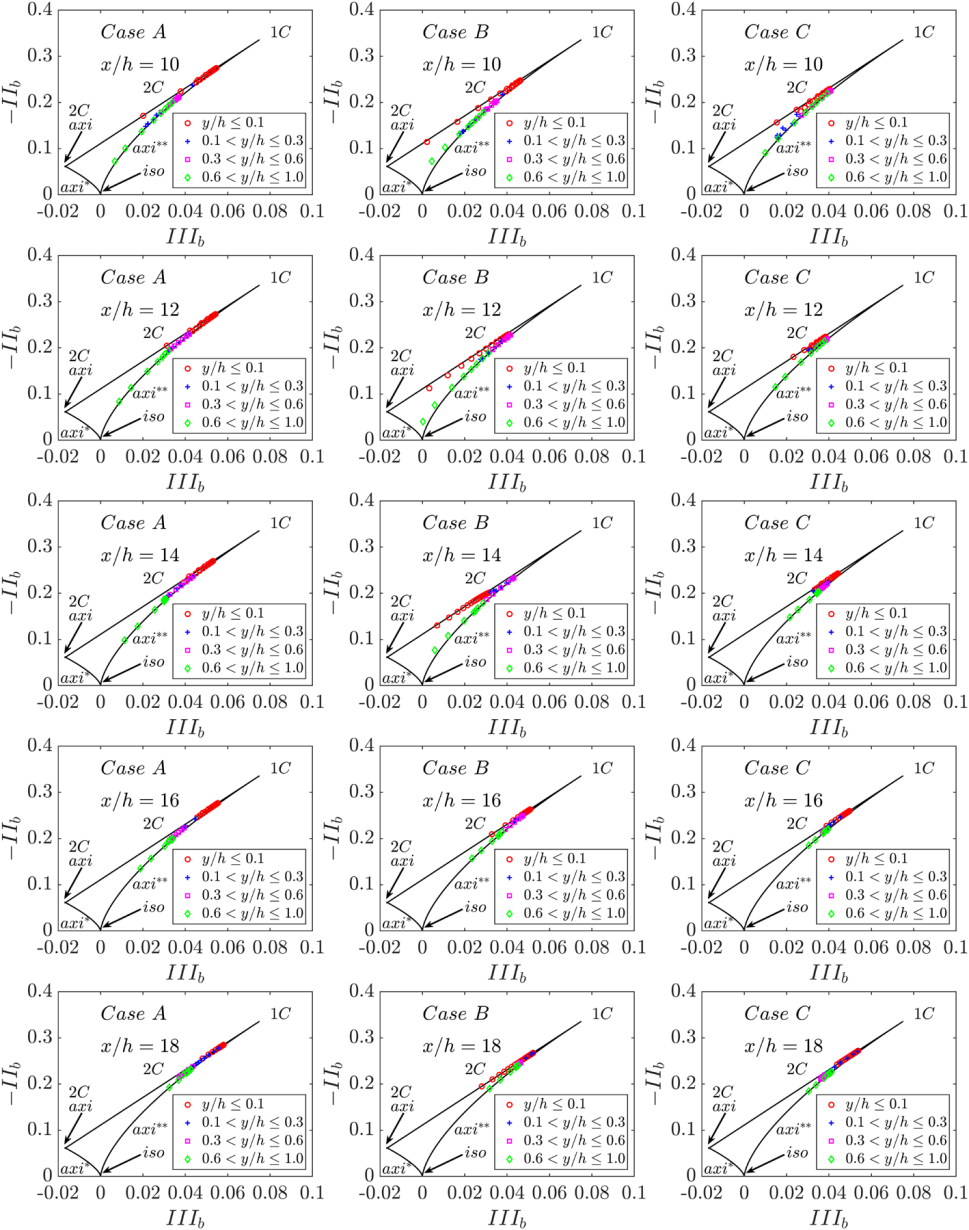
Plots of $$-I{I}_{b}$$ versus $$II{I}_{b}$$ in the form of the Lumley triangle for cases A-C (1st -3rd columns) at $$x/h= 10, 12, \text{14,16}$$ and $$18$$ (1st -5th row). Note $$ax{i}^{*}$$ represents axisymmetric contraction and $$ax{i}^{**}$$ represents axisymmetric expansion

The plots of -$I{I}_{b}$ vs. $II{I}_{b}$ are shown within the Lumley triangle in Fig. 14. It can be seen from Fig. 14 that the 2 component-elliptical limit (i.e., $\widetilde{k}$ is dominated by $\widetilde{{u}^{{\prime}{\prime}}{u}^{{\prime}{\prime}}}$ and $\widetilde{{w}^{{\prime}{\prime}}{w}^{{\prime}{\prime}}}$ contributions) is obtained close to the wall before approaching the 1-component limit for small values of $y/h$ (due to the peak value of predominant $\widetilde{{u}^{{\prime}{\prime}}{u}^{{\prime}{\prime}}}$ at small values of $y/h$) following that anisotropy tensor exhibits axisymmetric expansion before tending towards isotropy close to the centreline (i.e., $y/h\approx 1.0$ in these cases) for the corresponding non-reacting flow case. The 2-component-elliptical and axisymmetric expansion limits are mostly obtained during FWI, and thus Reynolds stress tensor becomes highly anisotropic during FWI. However, case C exhibits greater anisotropy compared to both case A and case B. The findings from Fig. 14 suggest that the thermal wall boundary conditions play a key role in determining the anisotropy of the Reynolds stress tensor. The extent of anisotropy plays a key role in the modelling of the pressure-strain correlation term in the closure of the Reynolds stress $\widetilde{{u}_{i}^{{\prime}{\prime}}{u}_{j}^{{\prime}{\prime}}}$ transport equation. The findings from Fig. 14 indicate that the influence of wall boundary conditions needs to be accounted for in the closures of the pressure-strain correlation term in the $\widetilde{{u}_{i}^{{\prime}{\prime}}{u}_{j}^{{\prime}{\prime}}}$ transport equation for the analysis of FWI within turbulent boundary layers.

**Fig. 15:**
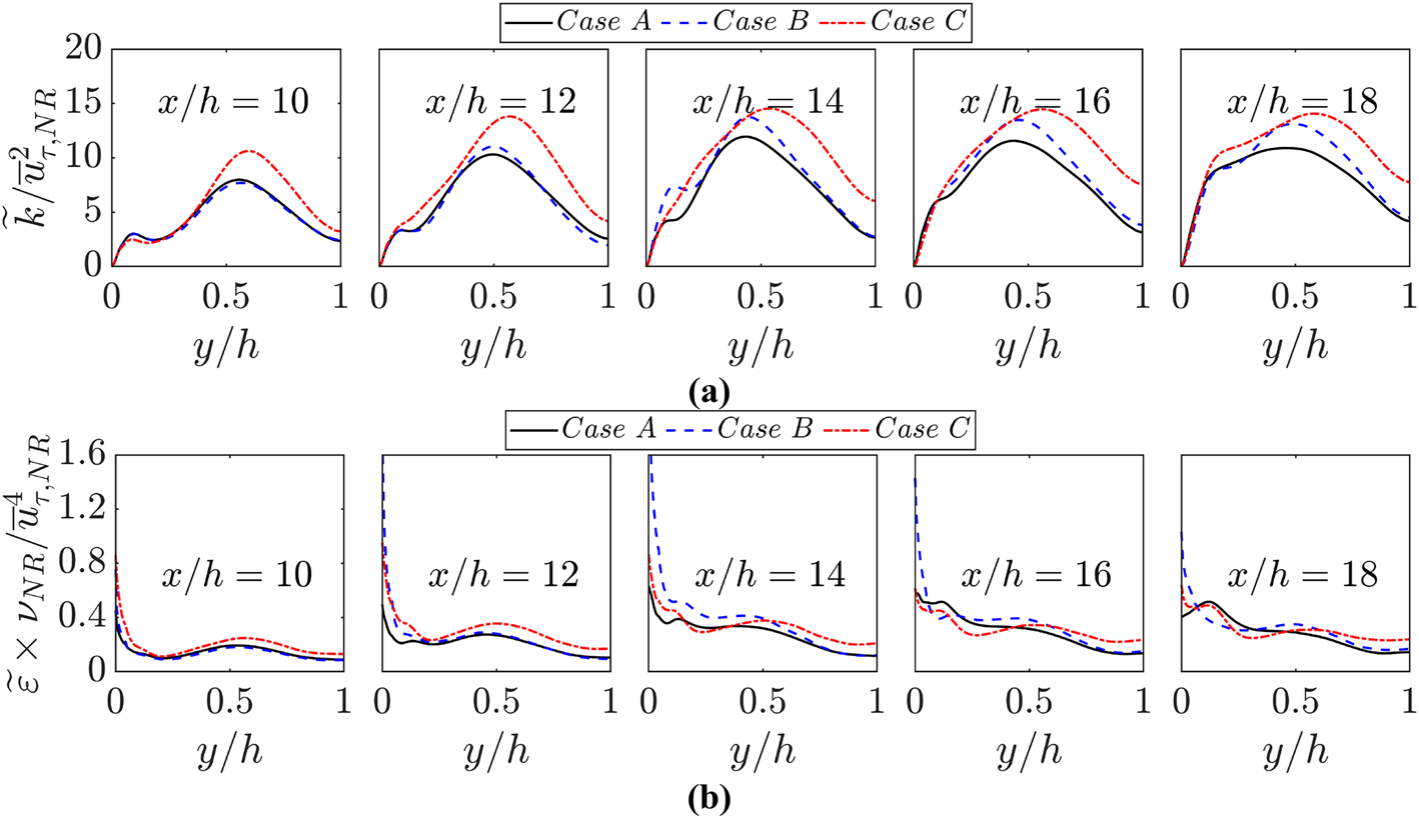
Variations of **a** $\widetilde{k}/{\overline{u} }_{\tau ,NR}^{2}$ and (b) $\widetilde{\varepsilon }\times {\nu }_{NR}/{\overline{u} }_{\tau ,NR}^{4}$ with $y/h$ at $x/h= 10, 12, \text{14,16}$ and $18$ for cases A-C

The greater (smaller) magnitudes of leading diagonal components of the Reynolds stress tensor in case C (case A) yields the highest (smallest) value of $\widetilde{k}=\widetilde{{u}_{i}^{{\prime}{\prime}}{u}_{i}^{{\prime}{\prime}}}/2$ during the FWI among all cases considered here, which can be substantiated by Fig. 15a, where the variations of $\widetilde{k}/{\overline{u} }_{\tau ,NR}^{2}$ with the normalised wall normal distance $y/h$ are shown at different streamwise distances. It has been demonstrated previously (Veynante et al. 1997) that a counter-gradient transport occurs when $\tau {S}_{L}/\sqrt{2\widetilde{k}/3}\gg 1$, whereas gradient transport is obtained for $\tau {S}_{L}/\sqrt{2\widetilde{k}/3}\ll 1$. The higher values of $\widetilde{k}$ give rise to smaller values of $\tau {S}_{L}/\sqrt{2\widetilde{k}/3}\ll 1$ in case C than in cases A and B and thus the propensity of gradient behaviour is stronger in case C than in cases A and B. Moreover, smaller values of $\widetilde{k}$ lead to higher values of $\tau {S}_{L}/\sqrt{2\widetilde{k}/3}\ll 1$ in case A than in cases B and C and thus the propensity of counter-gradient behaviour is stronger in case A than the other cases considered here (see Figs. 13 and 14).

For the purpose of completeness, the variations of the normalised dissipation rate of turbulent kinetic energy $\widetilde{\varepsilon }\times {\nu }_{NR}/{\overline{u} }_{\tau ,NR}^{4}$ in the wall normal direction are shown in Fig. 15b, which shows that $\widetilde{\varepsilon }\times {\nu }_{NR}/{\overline{u} }_{\tau ,NR}^{4}$ drops from the wall with increasing $y/h$, and the same qualitative behaviour is observed for all cases. However, the stronger fluctuating velocity gradient owing to augmented effects of thermal expansion gives rise to higher values of $\widetilde{\varepsilon }=\overline{\mu (\partial {u}_{i}^{{\prime}{\prime}}/\partial {x}_{j})(\partial {u}_{i}^{{\prime}{\prime}}/\partial {x}_{j})}/\overline{\rho }$ in cases B and C than in case A. The values of $\widetilde{k}/{\overline{u} }_{\tau ,NR}^{2}$ and $\widetilde{\varepsilon }\times {\nu }_{NR}/{\overline{u} }_{\tau ,NR}^{4}$ drop with the progress of FWI which is consistent with previous findings by the present authors and the physical explanations for these behaviours are provided elsewhere (Ahmed et al. 2023; Ghai et al. 2023a).

### Modelling Implications

The results shown in Figs. 6, 7, 8 and 9 indicated that the wall boundary condition does not affect the statistical behaviour of scalar variances, scalar dissipation rate and scalar fluxes away from the wall but the near-wall behaviour during FWI changes. Moreover, the wall temperature does not significantly alter the qualitative behaviours of scalar variances and dissipation rates of reaction progress variable and non-dimensional temperature but the near-wall behaviour of these quantities in the case of adiabatic wall case are notably different to those in the case of isothermal walls due to the absence of flame quenching for adiabatic walls. Most existing models for scalar variances and dissipation rates for premixed combustion have been proposed and benchmarked for adiabatic conditions, so these models might be rendered invalid for isothermal wall boundary conditions. However, the qualitative similarity of scalar variances and dissipation rates of reaction progress variable and non-dimensional temperature for different wall temperatures for isothermal walls indicate that the models proposed for isothermal wall boundary condition for a given wall temperature are likely to be valid for isothermal boundary condition corresponding to other temperatures.

The differences in density and kinematic viscosity at the wall yield different values of ${y}^{+}$ and ${u}^{+}$ for a given wall normal distance when different wall boundary conditions are used, although $\widetilde{u}$ variations with $y$ remain comparable. This has implications on the extent of deviations from the log-law depending on the thermal wall boundary condition although the log law for ${u}^{+}$ with ${y}^{+}$ has been found to be invalid for all cases considered here. This implies that the performance of Reynolds Averaged Navier–Stokes (RANS) turbulence models relying on log-law based wall functions can yield different levels of accuracy in response to different thermal wall boundary conditions. Moreover, the magnitudes of Reynolds stresses, kinetic energy and its dissipation rate have been found to increase with increasing wall temperature for isothermal wall boundary conditions although the qualitative behaviours in terms of spatial distribution and anisotropy of Reynolds stress tensor remain unaffected. This implies that the Reynolds stress closure in FWI is likely to be affected by the thermal boundary condition and the conventional Boussinesq’s hypothesis for Reynolds stress closure might be rendered invalid during FWI for the cases considered here irrespective of thermal wall boundary condition. The differences in the magnitude of turbulent kinetic energy in response to thermal wall boundary condition also affect the nature of turbulent scalar flux behaviour. Thus, thermal boundary condition could also indirectly affect the closures of turbulent scalar fluxes.

## Conclusions

The effects of thermal wall boundary condition in the oblique flame–wall interaction of V-shaped premixed flames in a channel flow configuration have been analysed using 3D DNS data corresponding to a friction Reynolds number of $R{e}_{\tau }=110$. Three different wall boundary conditions have been considered for the analysis: (1) isothermal chemically inert wall where the wall temperature is the same as the unburned gas temperature; (2) adiabatic inert wall; (3) isothermal wall but the wall temperature is higher than the unburned gas temperature. The main findings can be summarised in the following manner:

- It has been found that the flame angle is the largest for the elevated wall temperature case and the smallest for the isothermal wall case with the wall temperature that of the unburned gas mixture. This suggests that the flame intersection with the wall occurs at a distance, which is the smallest for the elevated wall temperature case and the highest for the isothermal wall at the unburned gas temperature. This suggests that at a given streamwise location, the FWI remains at the more advanced stage for the elevated wall temperature isothermal wall case than the isothermal cold wall case.
- The flame does not quench in the case of the adiabatic wall but the flame quenches due to heat loss through the wall in the isothermal wall cases. The minimum flame quenching distance decreases and the maximum wall heat flux magnitude increases significantly with increasing wall temperature for the isothermal boundary condition.
- It has been found that the wall temperature for isothermal wall cases does not significantly alter the qualitative nature of the statistical behaviours of scalar variances and dissipation rates of reaction progress variable and non-dimensional temperature. However, the near-wall behaviour of these quantities in the case of adiabatic wall have been found to be signficantly different to those in the case of isothermal walls owing to the absence of flame quenching in the case of adiabatic walls.
- Upstream of the flame–wall interaction, the mean friction velocity values are significantly higher when the wall temperature is elevated for the isothermal wall compared to both the isothermal wall at reactant temperature and the adiabatic wall cases. This is a consequence of the greater magnitude of velocity gradient induced by stronger thermal expansion effects in in isothermal case with elevated wall temperature. During the flame–wall interaction, the mean friction velocity values decrease for both scenarios involving isothermal walls. By contrast, for adiabatic wall condition, the mean friction velocity initially rises, followed by a subsequent decrease in values. However, these values persist at a level exceeding those observed in both cases with isothermal wall boundary condition. The differences in density at the wall due to the thermal boundary condition give rise to significant differences in the friction velocity behaviour. The differences in density and kinematic viscosity at the wall give rise to different values of ${y}^{+}$ and ${u}^{+}$ for a given wall normal distance for different boundary conditions although $\widetilde{u}$ values at a given wall-normal distance remain comparable for all cases considered here.
- The log-law does not capture the variation of ${u}^{+}$ with ${y}^{+}$ obtained from DNS data for the different boundary condition cases considered here. However, the extent of deviation of the variation of ${u}^{+}$ with ${y}^{+}$ from log-law is likely to be different for different thermal wall boundary conditions.
- An increase in wall temperature acts to increase the magnitudes of Reynolds stresses, kinetic energy and its dissipation rate with an increase in wall temperature for isothermal wall boundary condition but the qualitative behaviours in terms of spatial distribution and anisotropy of Reynolds stress tensor remain unaffected.
- The Boussinesq hypothesis does not predict the correct sign of the Reynolds shear stress during the early stages of flame–wall interaction, but this hypothesis predicts the correct sign of the Reynolds shear stress at the later stages of flame–wall interaction when the effects of thermal expansion are weak.
- The effects of thermal wall boundary condition on turbulent kinetic energy and Reynolds stresses indirectly influence the statistical behaviour of turbulent scalar flux components. The scalar flux shows counter-gradient behaviour in the regions of intense heat release during the early stages of flame–wall interaction, but this tendency weakens at later stages of flame–wall interaction.

The above findings have implications in the context of closures of scalar variance, dissipation rate, Reynolds stress and turbulent scalar flux. Thus, thermal wall boundary condition effects need to be included in these closures for flame–wall interaction. As the present analysis focuses principally on turbulence and scalar fluctuation statistics, the simplification of chemistry is unlikely to affect the qualitative nature of the results reported here but further analysis is needed in the presence of detailed chemistry for higher values of $R{e}_{\tau }$ in order to obtain deeper understanding. This will form the platform for further analyses.

## Data Availability

No datasets were generated or analysed during the current study.
